# Nudging customers towards healthier food and beverage purchases in a real-life online supermarket: a multi-arm randomized controlled trial

**DOI:** 10.1186/s12916-021-02205-z

**Published:** 2022-01-17

**Authors:** Josine M. Stuber, Jeroen Lakerveld, Loes W. Kievitsbosch, Joreintje D. Mackenbach, Joline W. J. Beulens

**Affiliations:** 1 Department of Epidemiology and Data Science, Amsterdam Public Health Research Institute, Amsterdam UMC, VU University Amsterdam, De Boelelaan 1117, 1081 HV Amsterdam, Netherlands; 2 Upstream Team, www.upstreamteam.nl, Amsterdam UMC, VU University Amsterdam, De Boelelaan 1117, 1081 HV Amsterdam, Netherlands; 3 Julius Center for Health Sciences and Primary Care, University Medical Center Utrecht, Utrecht University, Universiteitsweg 100, 3584 CG Utrecht, Netherlands

**Keywords:** Choice architecture, Food environment, SES, Health inequalities, Public health

## Abstract

**Background:**

Nudging is increasingly used to promote healthy food choices in supermarkets. Ordering groceries online is gaining in popularity and nudging seems efficacious there as well, but is never comprehensively tested in real-life. We evaluated the real-life effectiveness of nudging in an online supermarket on healthy food purchases.

**Methods:**

We conducted a multi-arm, parallel-group, individually randomized controlled trial in an online supermarket. During 1 month, all customers were randomized to (1) control condition, (2) information nudges, (3) position nudges, and (4) information and position nudges combined. Allocation was concealed and customers were not blinded, but unaware of the intervention. Mean differences between the control condition and the intervention arms in the total percentage of healthy purchases were assessed with a linear mixed model. We tested for effect modification by area-level deprivation.

**Results:**

Based on sales data from 11,775 shoppers, no overall significant effects were detected. Yet, effects were modified by area-level deprivation (*p*_Arm 2_ < 0.001). Among shoppers from deprived areas, those allocated to information nudges purchased a 2.4% (95%CI 0.8, 4.0) higher percentage of healthy products compared to controls. No significant differences were observed for position (− 1.3%; 95%CI − 2.8, 0.3) and combined nudges (− 0.1%; 95%CI − 1.7, 1.5). Shoppers from non-deprived areas exposed to information nudges (− 1.6%; 95%CI − 3.2, − 0.1) and the combined nudges (− 2.1%; 95%CI − 3.6, − 0.6), but not position nudges (− 0.9%; 95%CI − 2.4, 0.7), purchased a lower percentage of healthy products.

**Conclusion:**

Information nudges in an online supermarket can increase healthy product purchases, but only for those living in deprived areas. The adverse effects found on purchasing behaviors for those from non-deprived areas call for further research. Further research should also focus on real-life effects of online healthy food nudging as part of a broader nutrition intervention strategy, and on the equitability of the online nudging intervention within populations.

**Trial registration:**

Retrospectively registered in the ISRCTN registry at May 21, 2021 (ISRCTN10491616).

**Supplementary Information:**

The online version contains supplementary material available at 10.1186/s12916-021-02205-z.

## Background

Cardiovascular diseases and type 2 diabetes are a major burden of disease worldwide. Unhealthy dietary intake is an important modifiable risk factor for chronic diseases [1]. Additionally, there are persistent socioeconomic inequalities in chronic disease risk. Those with a lower socioeconomic position (SEP) tend to have unhealthier dietary patterns—putting them at increased risk [2]. As such, addressing unhealthy dietary patterns is crucial in order to reduce chronic disease burden and inequalities therein.

Nudges can be used to promote healthy dietary choices. Nudges target automatic food choices which do not require high levels of individual agency to change behaviors. This is in contrast to strategies targeting deliberate food choices [3], which require high levels of self-efficacy and motivation. Individual-level interventions targeting these deliberate choices often have limited and non-sustained effects, especially in those who rely on fewer resources such as populations with a lower SEP [4]. Nudging as a low-agency population-level intervention has the potential to make healthy dietary choices easier, and is likely more equitable [4]. Evidence from real-life settings suggests that nudging can help in promoting healthier purchases [5].

The typology of interventions in proximal physical micro-environments (TIPPME) typology [6] classifies nudges into those targeting the *placement* and the *properties* of products. *Placement* refers to the availability and the positioning of products. *Properties* refer to the functionality or design of products or highlight specific product information (e.g., product labeling). It is, however, likely that for nudging to have a substantial impact on dietary patterns, multiple types of nudging strategies need to be combined across various food groups to overcome habitual purchasing behaviors.

Supermarkets are an important setting for nudging interventions as—in Europe—80% of all foods and beverages are purchased there. Nowadays, online grocery services are increasingly popular and offer opportunities for supermarkets to promote healthier food purchases on a large scale and at relatively low costs. Tentative evidence suggests that information nudges [7–16], placement nudges [17–21], or a combination of placement and availability nudges [22], are efficacious in promoting healthier purchases in an online supermarket setting. Only one study, testing healthier swap suggestions via online pop-ups, did not report beneficial effects on purchasing behaviors [23].

However, all these studies used simulated settings where participants were not actual customers spending their grocery budget. Real-life effects are likely attenuated compared to results from these simulated settings [24]. For example, for in-store product labeling approaches it was estimated that effect sizes are up to 17 times smaller when study participants are real customers [24]. To the best of our knowledge, one real-life study comparing one intervention with one control store investigated the effect of an online information nudge. This information nudge used on milk, bread, breakfast cereals, biscuits, and frozen meals revealed no effects on sales data [25]. Altogether, there is very little real-life evidence on the effect of nudges across food groups in an online setting.

In the present study, we therefore implemented a single and combined nudging intervention in an online supermarket and evaluated the real-life effectiveness on the total percentage of healthy products purchased (primary aim). In addition, we conducted exploratory analyses with secondary (effectiveness of nudging strategies across individual food groups) and tertiary (total retailer revenue) outcomes. Finally, we explored whether any effects were modified by area-level deprivation.

## Methods

### Trial design

As part of the Supreme Nudge project [26], we evaluated the effectiveness of two nudging strategies to promote healthy food and beverage purchases in a Dutch online supermarket, using a multi-arm, parallel-group, individually randomized controlled trial (RCT).

- Arm 1: regular online supermarket used as control condition;
- Arm 2: information nudges;
- Arm 3: position nudges;
- Arm 4: information and position nudges.

This multi-arm design allowed for disentangling of the single and combined effects of both nudging types. The reporting here follows the extension of the CONSORT statement for multi-arm trials [27].

### Participants

The trial was implemented in a Dutch online supermarket chain for five consecutive weeks, between mid-August and mid-September 2020. During this period, sales data were collected from all customers who placed a delivery order. Collected customer sales data included the number of items, the weight, and the price (Euros) of each product purchased. As each online order was connected to a physical supermarket for delivery, data indicated the supermarket location. Additionally, data described whether it concerned a private or business-related order, and included the customers’ self-reported sex and year of birth. The latter was used to calculate age. Finally, sales data contained customers’ 4-digit postal codes used to determine area-level deprivation, based on area-level status scores [28]. The status scores constituted of average level of education, income, and employment. Four-digit postal codes consist of 2250 addresses on average. We classified all customers into living in a deprived area (status score below the national average) or a non-deprived area (score at or above the national average). We aimed to include shoppers reflecting an average household grocery shopping pattern. Therefore, business-related customers and customers whose purchases consisted for > 90% of alcohol, unhealthy other foods, or snacks were excluded from the analysis.

### Interventions

The information nudge was implemented on healthy products and highlighted specific product information [6]. It was developed following a co-creation process with the supermarket chain, as part of the Supreme Nudge supermarket trial [29, 30]. Consequently, the layout of the information nudge corresponded with the supermarket chain’s corporate identity. The content focused on positive ways to stimulate healthy purchases, without specifically stating that the targeted products are healthy*.* This approach was based on a qualitative study among the lower SEP target group of the supermarket trial exploring the perceptions of supermarket nudging interventions. Results reveal that participants preferred nudges that are in line with for example preferred taste and preparation time, and some expressed distrust and suspected ulterior motives towards supermarket product labels which presented nutrition information or specially stated *healthy* [31].

The information nudge consisted of an overarching theme (*‘Lots of choice... We are happy to help!’*), which introduced three types of product labels. They highlighted a product’s *tastiness*, *convenience*, or *popularity*. A banner on the home webpage and each product category page explained the nudging theme (Additional file 1: Supplementary Figs. 1 and 2). All three labels were simultaneously included in the intervention arms containing the information nudge. A researcher discussed and decided with supermarket employees which product groups were assigned to each of the nudging themes. For example, all healthy canned fish received the *convenience* label, all healthy cheeses the *tastiness* label, and all fresh vegetables the *popularity* label (Additional file 1: Supplementary Table 1).

The position nudge increased the number of healthy product placements [6]. Placement of the healthy alternatives was discussed with supermarket e-commerce employees and resulted in two feasible components. First, unhealthy-to-healthy product swaps were suggested when viewing a comparable unhealthy product (e.g., four wholegrain bread options on a white bread product page). They were displayed at the bottom of the product page, introduced as ‘*Also frequently purchased by other customers*’ (Additional file 1: Supplementary Fig. 3). Second, the check-out page suggested four standardized healthy products, introduced as ‘*Tasty alternatives*’ (Additional file 1: Supplementary Fig. 4).

The nudging strategies focused on healthy food groups as recommended within the food-based Dutch Dietary Guidelines (including fruits, vegetables, fiber-rich products, healthy fats, and non-sugary beverages) [32]. Based on these guidelines, the Netherlands Nutrition Centre has composed a data base with all supermarket food and beverage products and whether they are recommended within a healthy diet [33]. Using this database, we categorized all online supermarket products into 19 relevant food groups, within which we divided products into healthy and unhealthy (Table 1). As customers accessed an online supermarket connected to a store location in their neighborhood, the proportion of healthy versus unhealthy products was similar across stores but the absolute number of available products in the online supermarket varied (range 6000–14,336). All non-food products and baby foods were excluded from the food-group categorization, leaving a maximum of 9217 products available (Table 1).

**Table 1:** Total number of nudged and non-nudged products available in the online supermarket

	Healthy products		Unhealthy or neutral products	
	Information nudges	No intervention	Position nudges	No intervention
**All foods and beverages** **(*n*** **= 9217)**	*n*=966; 62% of all healthy products	*n*=604	*n*=486; 6% of all unhealthy products	*n*=7161
**Fruits (*n*=211)**	Fresh, pre-cut, and frozen fruits (*n*=94; 61% of all healthy fruits)	Fresh fruit, canned fruits, apple sauce (*n*=61)	N/A	Canned fruits with added sugar, apple sauce with added sugar, fruit puree with added sugar (*n*=56)
**Vegetables (*n*=415)**	Fresh, pre-cut, and frozen vegetables (*n*=199; 73% of all healthy vegetables)	Fresh vegetables (*n*=75)	N/A	Vegetables with added cream, canned vegetables (salted) (*n*=141)
**Breads (*n*=218)**	Wholegrain bread (*n*=57; 90% of all healthy bread)	Wholegrain bread (*n*=6)	White and non-wholegrain brown bread (*n*=61; 39% of all unhealthy bread)	White and non-wholegrain brown bread (*n*=94)
**Bread substitutes (*n*=222)**	Wholegrain crackers, oats, muesli (*n*=28; 88% of all healthy bread substitutes)	Oats, muesli (*n*=4)	Non-wholegrain crackers, breakfast cereals with added sugar, salt, and/or fat (*n*=142; 75% of all unhealthy bread substitutes)	Rice crackers, rusk, non-wholegrain crackers, breakfast cereals with added sugar, salt, and/or fat (*n*=48)
**Potatoes (*n*=105)**	N/A	Fresh and/or pre-cut unprocessed potatoes (*n*=50)	N/A	Processed potatoes (*n*=55)
**Pasta and rice (*n*=208)**	Wholegrain pasta and rice (*n*=28; 88% of all healthy pasta and rice)	Wholegrain pasta and rice (*n*=5)	Non-wholegrain pasta and rice (*n*=103; 59% of all unhealthy pasta and rice)	Non-wholegrain pasta and rice (*n*=72)
**Teas and coffees (*n*=361)**	Tea bags (*n*=102; 35% of all healthy tea and coffee)	Tea bags, filtered coffee products (*n*=190)	N/A	Unfiltered coffee products, tea bags (added sugar) (*n*=69)
**Sodas, waters, and juices (*n*=735)**	Water, flavored water (unsweetened) (*n*=67; 96% of all healthy beverages)	Flavored water (unsweetened) (*n*=3)	N/A	Sodas, energy drinks, fruit juices, lemonade (*n*=665)
**Cheeses (*n*=332)**	Low-fat and low-salt cheeses (*n*=28; 72% of all healthy cheeses)	Low-fat and low-salt cheeses (*n*=11)	N/A	High-fat and/or high-salt cheeses (*n*=293)
**Milk and yogurt products (*n*=571)**	Semi-skimmed and skimmed milk and yogurt products (*n*=111; 71% of all healthy milk and yogurt)	Soy-dairy products (unsweetened), semi-skimmed and skimmed sterilized milk, coffee milk (*n*=46)	N/A	Semi-skimmed and skimmed dairy products (sweetened), full-fat dairy, custard, desserts, pudding, whipped cream, pudding, cooking cream, dairy drinks, chocolate milk, soy-dairy products (sweetened), ice cream (*n*=414)
**Meats (*n*=769)**	N/A	Unprocessed and low-fat meats, meat substitutes (unsalted), and eggs(*n*=105)	N/A	Processed and high-fat meats, and meat substitutes (salted) (*n*=664)
**Fish (*n*=140)**	Fresh, frozen, canned, and breaded fish (> 70% fish) (*n*=87; 92% of all healthy fish)	Canned fish (*n*=8)	N/A	Breaded fish (< 70% fish), processed fish dishes (*n*=45)
**Legumes (*n*=135)**	Canned legumes (low salt) (*n*=63; 94% of all healthy legumes)	Canned legumes (low salt) (*n*=4)	N/A	Canned legumes (high salt) (*n*=68)
**Nuts (*n*=179)**	Seeds, nuts, peanuts, natural peanut butter (*n*=34; 67% of all healthy nuts)	Seeds, nuts, peanuts, natural peanut butter (*n*=17)	Nuts, peanuts (salted) (*n*=103; 81% of all unhealthy nuts)	Sugared peanuts, peanut butter (added salt and palm oil) (*n*=25)
**Fats (*n*=163)**	Olive oils, sunflower oils, and margarines (*n*=68; 78% of all healthy fats)	Vegetable oils, frying oils (*n*=19)	Butters, baking butters (*n*=56; 78% of all unhealthy fats)	Frying oils, coconut oils, butters, baking butters (*n*=20)
**Other foods (*n*=2077)**	N/A	N/A	N/A	Ready-to-eat meals, meal salads, pancakes, pizza, canned soup, drinking broth, seasoning products (*n*=2077)
**Savory snacks (*n*=417)**	N/A	N/A	Savory snacks (*n*=21; 5% of all unhealthy snacks)	Salty snacks, fried snacks, chips, popcorn, bread sticks, (*n*=396)
**Sweet snacks (*n*=1255)**	N/A	N/A	N/A	Cookies, candy, confectionary, chocolate, liquorice, bubblegum, water-based ice cream (*n*=1255)
**Alcoholic drinks (*n*=704)**	N/A	N/A	N/A	All alcoholic drinks (*n*=704)

The implementation feasibility was discussed with the supermarket chain, resulting in that 62% of all healthy products received the information nudges. For the position nudges, the supermarket chain indicated that a limited amount of food groups could be targeted as supermarket staff needed to manually enter the unhealthy-to-healthy swaps to the system. We decided to target the breads, bread substitutes, pasta and rice, nuts, and fats, for which comparable within-food group swaps were available (e.g., sugary peanuts to unsalted peanuts). Consequently, position nudges were implemented on 6% of all unhealthy products. Regarding the healthy check-out suggestions, four standardized products were selected, consisting of two buckets of snack-sized vegetables and two boxes of dried fruits.

### Outcomes

The primary outcome on which study conclusions were based was the total percentage of healthy grams purchased, calculated based on the sum of healthy grams purchased relative to all grams purchased. We pre-determined a minimum of a 1% significant difference in the primary outcome as relevant difference, for two reasons. First, a small increase in overall healthier purchasing behaviors within individuals can have a substantial impact on healthier dietary behaviors when translated to population levels. Second, based on results seen in previous studies (e.g., [17]) it is realistic to expect that environmental changes in a supermarket setting to yield modest shifts in purchasing behaviors. As a secondary outcome, we conducted exploratory analyses to examine which food groups drove the overall differences and whether nudging strategies had differential effects across food groups. Therefore, we provide insight in the total percentage of healthy purchases within food groups calculated by the sum of healthy grams purchased within a specific food group, relative to all grams purchased within that food group. Last, in exploratory analyses with the tertiary outcome, we investigate the total retailer revenue (Euros) as relevant business-related outcome.

### Sample size

During the conception of this study, the supermarket chain indicated to have ~ 28,000 monthly grocery orders in their online supermarket. Based on an implementation duration of five weeks, we expected to collect data from ~ 5500 shoppers per trial arm. As simulated experimental studies on online nudging strategies relied on much smaller samples (e.g., ranging from 218 up to 476 per intervention arm [17, 18, 22]), we were confident to have secured adequate power for the analyses and considered a corresponding sample size calculation unnecessary.

### Implementation and randomization

E-commerce employees of the supermarket implemented the online nudging strategies. Blinding was not possible due to the nature of the intervention, but customers were not actively notified of the nudges. Randomization was concealed as supermarket employees conducted the randomization and allocation of customers to one of the trial arms in a software system called Blueconic – Customer Data Platform. The system was set to randomize and allocate the customers in equal distribution percentages of 25% across the four trial arms. Random allocation was based on Internet Protocol (IP-)addresses. Data on IP-addresses was not stored. As such, when customers re-visited the online supermarket, their IP-address was re-randomized and re-allocated to one of the arms. Consequently, customers could theoretically participate in the trial more than once and resulted in the sample to consist of shoppers rather than individual customers. Therefore, from now on, we refer to shoppers rather than customers.

### Statistical methods

Descriptive statistics were reported by the trial arm and consisted of the proportion of females (*n*(%)), the mean age (±standard deviation (SD)), the proportion of shoppers from deprived areas (*n*(%)), and the total amount of grams purchased per shopper (median and inter quartile range (IQR)). Moreover, for the control condition in arm 1, we reported the total percentage of healthy grams purchased, and per food group (mean and 95% confidence interval (CI)), as well as the absolute grams healthy and the grams unhealthy purchased per food group (mean (95% CI)).

A linear mixed model with a random intercept on the store level was used to assess the mean differences between arm 1 and the intervention arms in the percentage of all healthy purchases (primary outcome). Residual plots indicated adequate model fit. As the sales data of the exploratory analyses with secondary and tertiary outcomes were U-shaped and/or highly right skewed, we conducted nonparametric bootstrapping for hierarchical data to estimate the mean differences in purchases across individual food groups (secondary outcomes) and in total revenue (tertiary outcome) [34]. Bootstrap analyses were based on 10,000 non-parametric bootstrap replicates. Shoppers that did not purchase from a specific food group were excluded from the bootstrap analysis for that food group. Hence, the number of shoppers varied for each food group outcome. Statistical significance of all outcomes was defined as the absence of zero in the 95% CI.

We tested for effect-modification by area-level deprivation by adding an interaction term to the primary outcome model. All results were stratified in the case of a significant interaction term for at least one of the trial arms (*p* < 0.05). To explore if differences in percentages of healthy purchases resulted from differential purchases of healthy or unhealthy foods, absolute mean differences between arms in grams of healthy and unhealthy foods purchased within all food groups were reported as well as sensitivity analyses. We did not adjust for multiple testing, as our parallel design aimed to identify the single effects of both nudging types on purchasing behaviors as well as their combination on one primary outcome, (i.e., the total percentage of healthy purchases) [35]. Considering the numerous outcomes among the exploratory individual food group analyses (secondary outcomes), the robustness of our findings was explored in a sensitivity analysis in which we set the confidence interval at a 98% level. Analyses were conducted with R (version 3.6.1) using the packages *lme4* and *boot*.

## Results

During the five-week intervention period, 15,045 individual shoppers were randomized across the trial arms. After exclusions, 11,775 shoppers remained for analyses (Additional file 1: Supplementary Fig. 5). Among this study sample, approximately 65% was female, with a mean age of 56 (±17) years, and about half of the shoppers were from deprived areas (Table 2). These characteristics were equally balanced across study arms, as well as the number of shoppers per food group. However, not all food groups were purchased in similar amounts (Table 2). The most frequently purchased food groups were fruits, vegetables, milk and yogurt products, and meats (> 2100 shoppers per arm), whereas pasta and rice, fish, legumes, and nuts were the least frequently purchased food groups (< 800 shoppers per arm).

**Table 2:** Customer characteristics of shoppers and the number of shoppers within specific food groups (*n*_total_=11,775)

	Arm 1		Arm 2		Arm 3		Arm 4	
	(***n***=2992)		(***n***=2864)		(***n***=2976)		(***n***=2943)	
**Females,** ***n*** **(%)**^**a**^	1931	(64.5)	1847	(64.5)	1989	(66.8)	2018	(68.6)
**Age, mean (SD)**^**b**^	56.5	(17.4)	57.0	(17.6)	56.8	(17.5)	55.9	(17.4)
**Shoppers from deprived areas,** ***n*** **(%)**	1503	(50.2)	1466	(51.2)	1494	(50.2)	1423	(48.4)
**Total grams purchased, median (IQR)**	21,766	(17,679)	21,172	(17,576)	21,275	(16,440)	21,995	(17,626)
*Shoppers within food groups, n* (%)								
**Fruits**	2179	(72.8)	2100	(73.3)	2148	(72.2)	2120	(72.0)
**Vegetables**	2309	(77.2)	2235	(78.0)	2324	(78.1)	2292	(77.9)
**Breads**	1796	(60.0)	1761	(61.5)	1848	(62.1)	1811	(61.5)
**Bread substitutes**	1038	(34.7)	980	(34.2)	1011	(34.0)	977	(33.2)
**Potatoes**	1202	(40.2)	1114	(38.9)	1067	(35.9)	1151	(39.1)
**Pasta and rice**	773	(25.8)	795	(27.8)	784	(26.3)	832	(28.3)
**Teas and coffees**	1163	(38.9)	1213	(42.4)	1193	(40.1)	1148	(39.0)
**Sodas, waters and juices**	1955	(65.3)	1828	(63.8)	1914	(64.3)	1939	(65.9)
**Cheeses**	1759	(58.8)	1745	(60.9)	1739	(58.4)	1778	(60.4)
**Milk and yogurt products**	2623	(87.7)	2493	(87.0)	2590	(87.0)	2572	(87.4)
**Meats**	2558	(85.5)	2465	(86.1)	2556	(85.9)	2463	(83.7)
**Fish**	721	(24.1)	660	(23.0)	683	(23.0)	701	(23.8)
**Legumes**	520	(17.4)	569	(19.9)	537	(18.0)	574	(19.5)
**Nuts**	779	(26.0)	805	(28.1)	846	(28.4)	846	(28.7)
**Fats**	1502	(50.2)	1480	(51.7)	1488	(50.0)	1457	(49.5)
**Other foods**	2526	(84.4)	2472	(86.3)	2552	(85.8)	2529	(85.9)
**Savory snacks**	1589	(53.1)	1510	(52.7)	1603	(53.9)	1613	(54.8)
**Sweet snacks**	2186	(73.1)	2057	(71.8)	2101	(70.6)	2098	(71.3)
**Alcoholic drinks**	1001	(33.5)	1002	(35.0)	1112	(37.4)	1078	(36.6)

^a^3 missing values; ^b^69 missing values

Shoppers in arm 1 from deprived areas purchased on average 36.1% (SD 22.2) healthy products per shop, whereas those from non-deprived areas purchased 40.1% (SD 21.9) healthy products (Additional file 1: Supplementary Table 2). Shoppers in arm 1 from non-deprived areas purchased a higher percentage of healthy products compared to shoppers from deprived areas within various food groups, such as vegetables (89.0% versus 85.7%), pasta and rice (19.6% versus 13.6%), and milk and yogurt products (54.0% versus 48.1%) (Additional file 1: Supplementary Table 2). Regarding the grams of healthy products purchased, shoppers in arm 1 from non-deprived areas purchased on average 377 grams of fruits, 492 grams of vegetables, and 680 grams of healthy milk and yogurt products more than shoppers from deprived areas (Additional file 1: Supplementary Table 3).

### Primary outcomes

**Fig. 1:**
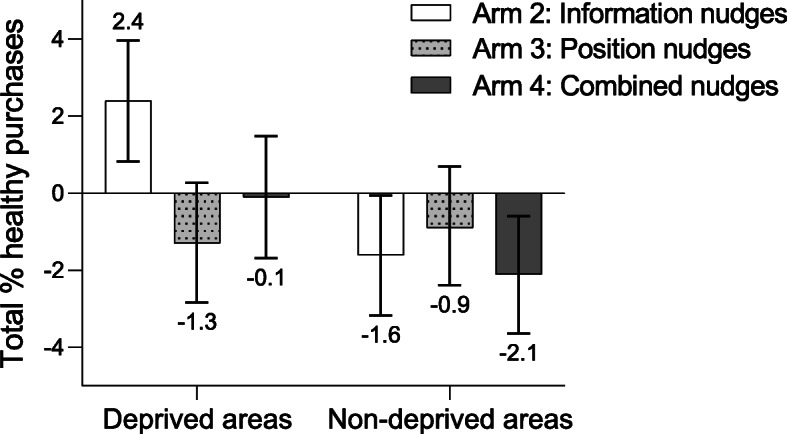
Mean differences in the total percentage of healthy purchases. Mean differences (95% CI bars) in the total percentage healthy purchases in Arm 2 (information nudge), Arm 3 (position nudge), and Arm 4 (information and position nudges) compared to arm 1, by area-level deprivation (*n*_total_=11,775)

No overall statistically significant intervention effects were observed for arm 2 (mean difference 0.4%; 95%CI − 0.7, 1.6), arm 3 (− 1.1%; 95%CI − 2.2, 0.0), nor arm 4 (− 1.1%; 95%CI − 2.2, 0.0). Yet, we found evidence for effect modification by area-level deprivation in Arm 2 (*p* < 0.001), but not in Arm 3 (*p* 0.741) and Arm 4 (*p* 0.057). The stratified results (Fig. 1) showed that among shoppers from deprived areas, those in arm 2 purchased a statistically significant 2.4% (95%CI 0.8, 4.0) higher percentage of healthy products compared to arm 1. No significant differences were observed in arm 3 (− 1.3%; 95%CI − 2.8, 0.3), nor in arm 4 (− 0.1%; 95%CI − 1.7, 1.5). For shoppers from non-deprived areas, those in arm 2 (− 1.6%; 95%CI − 3.2, − 0.1) and arm 4 (− 2.1%; 95%CI − 3.6, − 0.6) purchased a significantly lower percentage of healthy products, whereas this difference was non-significant in arm 3 (− 0.9%; 95%CI − 2.4, 0.7).

### Exploratory analyses on secondary outcomes

**Fig. 2:**
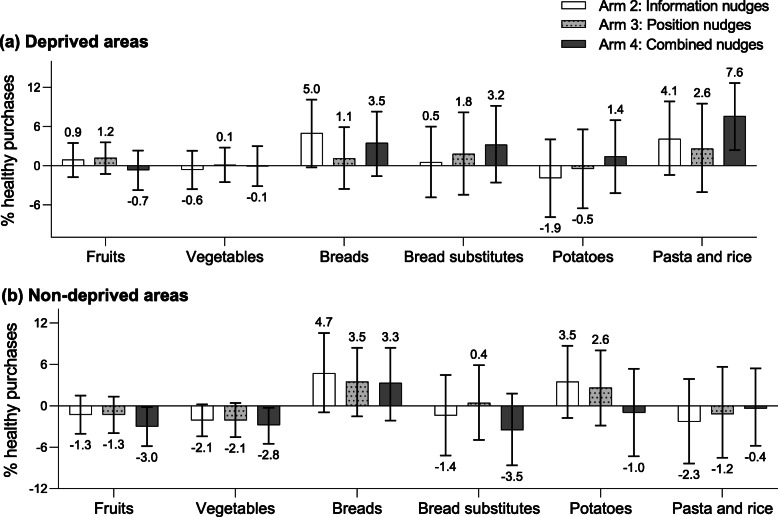
Mean differences in the percentage of healthy purchases within various carbohydrate products. Mean differences (95% CI) in the percentage of healthy purchases within various carbohydrate product groups in Arm 2 (information nudge), Arm 3 (position nudge), and Arm 4 (information and position nudges) compared to arm 1, for deprived areas (**a**) and non-deprived areas (**b**)

**Fig. 3:**
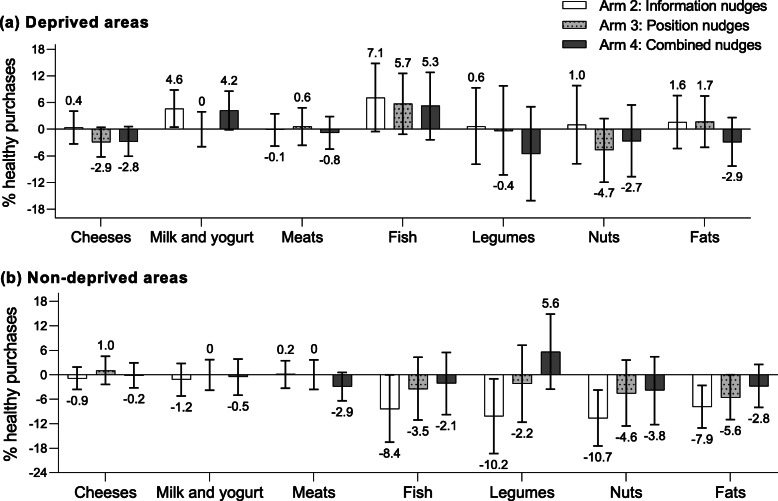
Mean differences in the percentage of healthy purchases within various protein and fat product groups. Mean differences (95% CI) in the percentage of healthy purchases within various protein and fat product groups in Arm 2 (information nudge), Arm 3 (position nudge), and Arm 4 (information and position nudges) compared to arm 1, for deprived areas (panel a) and non-deprived areas (panel b)

**Fig. 4:**
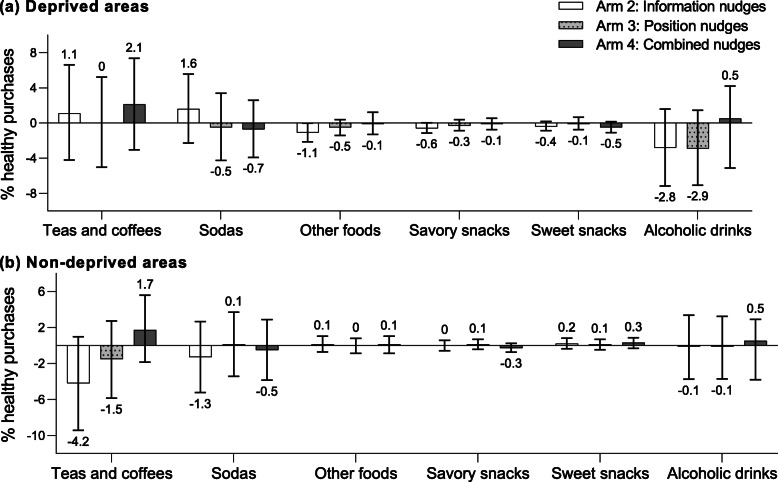
Mean differences in the percentage of healthy purchases within various beverage groups, other foods, and snacks. Mean differences (95% CI) in the percentage of healthy purchases within various beverage product groups, other foods and snacks in Arm 2 (information nudge), Arm 3 (position nudge), and Arm 4 (information and position nudges) compared to arm 1, for deprived areas (**a**) and non-deprived areas (**b**)

Figures 2, 3, and 4 show the between-arm mean differences in the percentage of healthy purchases for all food groups analyzed, compared to arm 1. A numerical overview of all food group outcomes is included as Supplementary Table 4 (Additional file 1).

Evaluation of differential effects of the nudging strategies across food groups revealed that healthy breads (5.0%; 95%CI − 0.3, 10.1), milk and yogurt products (mean difference 4.6%; 95%CI 0.5, 8.8), and fish (7.1%; 95%CI − 0.5, 14.8) mainly drove the overall higher percentage of healthy purchases among those from deprived areas in arm 2 (Figs. 2a and 3a) Although arm 4 did not show an overall higher percentage of total healthy purchases, the individual food group analysis showed a higher percentage of healthy pasta and rice purchased (7.6%; 95%CI 2.4, 12.7), as well as a higher percentage of healthy breads (3.5%; 95%CI − 1.6, 8.3), bread substitutes (3.2%; 95%CI − 2.6, 9.2), milk and yogurt (4.2%; 95%CI − 0.1, 8.6), and fish (5.3%; 95%CI − 2.4, 12.8) (Figs. 2a and 3a).

For shoppers from non-deprived areas, the decrease in the total percentage of healthy purchases in arm 2 were mainly driven by a lower percentage purchased from healthy fish (− 8.4%; 95%CI − 16.5, − 0.0), legumes (− 10.2%; 95%CI − 19.3, − 1.0), nuts (− 10.7%; 95%CI − 17.5, − 3.8), and fats (− 7.9%; 95%CI − 13.1, − 2.7) (Fig. 3b). For shoppers from non-deprived areas in arm 4, the total lower percentage of healthy purchases were mostly driven by a lower percentage purchased from healthy fruits (− 3.0%; 95%CI − 5.8, − 0.2), vegetables (− 2.8%; 95%CI − 5.5, − 0.3), and bread substitutes (− 3.5%; 95%CI − 8.6, 1.8) (Fig. 2b).

### Exploratory analyses on tertiary outcomes

Total retailer revenue was not affected following the implementation of nudges (Additional file 1: Supplementary Table 5). Shoppers from deprived areas spent 2.06 Euros (95%CI − 2.67, 6.94) more in arm 2, 3.17 Euros (95%CI − 1.57, 8.30) more in arm 3, and 1.71 Euros (95%CI − 2.46, 5.86) more in arm 4 compared to arm 1. Among shoppers from non-deprived areas, the mean differences in Euros spent compared to arm 1 were − 1.88 (95%CI − 6.50, 2.92), − 1.97 (95%CI − 6.91, 2.90), and 0.74 (95%CI − 4.07, 5.67) for arm 2, 3, and 4, respectively.

### Sensitivity analyses

Evaluating the mean differences in grams of healthy and of unhealthy purchases, the total percentage of healthy pasta and rice was higher among shoppers from deprived areas in intervention arm 4 due to a significantly lower purchase of unhealthy grams (mean difference − 274; 95%CI − 505, − 75), but not a higher purchase of healthy grams (− 1; 95%CI − 244, 231) (Additional file 1: Supplementary Table 6). Moreover, shoppers from deprived areas in intervention arm 2 (271 g; 95%CI 15, 560) and arm 4 (249 g; 95%CI 41, 452) purchased on average one portion of healthy vegetables more. For shoppers from non-deprived areas, a significantly lower amount of fruits (~ 350 g) was purchased in all three intervention arms. For purchases of fish and fats, the lower percentage of healthy purchases originated from lower amounts of healthy grams purchased of − 63 (95%CI − 135, 10) and − 83 (95%CI − 243, 51), respectively. The lower percentage of healthy nuts and legumes purchased originated from lower amounts of healthy grams purchased combined with lower amounts of unhealthy purchased (Additional file 1: Supplementary Table 6). The direction of results were comparable with the main analyses when exploring the robustness of our findings at a 98% CI level (Additional file 1: Supplementary Table 7 and 8).

## Discussion

This study provides evidence that information nudges to promote healthier purchasing behaviors in an online supermarket can affect healthy food purchases, although the effect seems specific to a neighborhood level of deprivation. Adverse effects were observed among shoppers from non-deprived areas, as information nudges were followed by a lower percentage of healthy purchases. Supermarkets are commercial parties in the food system which rely on financial stability. In this study, retailer revenue was not affected by the implementation of the online nudges, thus indicating that the commercial viability of this public health strategy is secured.

This novel randomized controlled trial in an online supermarket has several strengths such as its large sample of customer-level purchasing data and implementation across the Netherlands securing high external validity. Generalizability of findings was enhanced via the real-life nature of this study, since customers made real purchases. Customers of the online supermarket are not subjected to external influences as in a physical supermarket, which resulted in homogeneous nudging exposure across all customers. Last, a large share of the supermarket assortment was targeted including various types of healthy products and product brands. A limitation of this study is the relative short follow-up time leaving potential long-term effects of online nudges unknown. Customers may need more time to become accustomed with nudges to overcome habitual purchasing habits. Moreover, we did not have access to the number of page views or clicks on certain products. Insight in these factors could have provided additional information on potential nudge effectiveness. The study design is limited to measuring between-subject differences, whereas measuring within-subject changes over time would strengthen the robustness of findings. In addition, the fact that shoppers could theoretically participate multiple times in the RCT in different trial arms may have attenuated nudging effects due to confusion on the changing online supermarket environment or the lack of repeated nudging exposure. Finally, we were unable to include individual-level SEP measures, but due to the small size of the 4-digital postal codes, area-level deprivation served as a good proxy.

The effect modification by area-level deprivation is an important finding of this study. It is known that individuals with a lower SEP generally have unhealthier dietary behaviors [2], which is also visible in the shopper patterns of our control arm. Consequently, there is greater potential for improvement of dietary behaviors among those from deprived areas—which may partly explain the more beneficial effects seen among these customers. Additionally, as the information nudge was specifically developed based on the needs and preferences of a population with a lower SEP, it is not surprising they yield larger effects among customers from deprived areas. On the other hand, explanations for the adverse effects observed among customers from non-deprived areas remain speculative. One study on an information nudge did not show moderating effects by SEP [22], while others indicated that groups with a higher SEP can show reactance following social norms and/or information nudges [36] and likely prefer to maintain autonomous choices [37]. Also, higher levels of health-consciousness could result in more deliberate food choices, instead of relying on automatic product choices [3], i.e., it may be customers with a higher SEP who experienced the information nudges as patronizing, threatening their autonomy, causing them to reject nudged products.

Another important finding of our study is the much smaller real-life effects than those seen in simulation studies. For example, a position nudge in a simulation study resulted in 23% more purchases of the targeted lower energy dense products [22]. However, determining how much smaller effect sizes exactly are is not straightforward due to the variety in outcome measures used, such as amounts of fiber or salt purchased [14, 18] or an improvement in diet quality [10]. Yet, it is notable that all of the aforementioned efficacy studies reported beneficial effects of online nudges [7–14, 17–22, 38, 39], except the one RCT testing healthier swap suggestions via pop-ups [23]. Correspondingly, our position nudge with unhealthy-to-healthy product swaps also seemed not able to affect purchasing behaviors on its own. Moreover, the food group analyses indicated that when combined with the information nudge did the healthy swaps seem to result in a higher percentage of healthy purchases of bread and pasta and rice. However, we conducted a large number of exploratory analyses regarding the food group analysis, and findings are exploratory which warrant further investigation. Yet, results seemed to suggest differential effects of nudges across product groups, and the potential effectiveness of nudging interventions on milk and yogurt, and grain products is somewhat surprising. Literature suggests that such staple food purchases are strongly based on habitual behaviors which are challenging to influence by nudging [40]. However, similar large effects of nudging interventions on dairy and grain products were demonstrated in a previous simulation study of our own [41]. Whereas comparisons of effects across study settings are not straightforward, it has been advocated that for nudging to have an impact on purchasing behavior in real-life settings it should be incorporated into a broader strategy, e.g., including pricing strategies. Their real-life effects combined with nudging in an online environment are however unknown and require further research. Effects may be inherently different from a physical store. For example, customers seem less price-sensitive and more brand-loyal in an online environment [42].

Online supermarkets will likely have a sustained increasing reach among the general population, as their availability and use is gaining popularity. Therefore, implementing online healthy food nudging strategies hold the potential to affect population diet quality. Future studies should however investigate potential adverse effects among customers with a higher SEP and test effects of other nudging combinations (e.g., using personalized labeling and feedback approaches) to design an optimal combination of interventions. It is plausible that such a broader strategy which, for example, combines online nudges and pricing strategies across multiple food groups, will yield small but relevant effects on purchasing behaviors to promote healthier population dietary behaviors. Such results are needed to adequately inform public health policy makers to implement equitable nutrition interventions with a wide reach among populations and reduce diet-related chronic disease risk.

## Conclusion

Information nudges in an online supermarket can increase healthy product purchases, but only among those living in deprived areas. Commercial viability for the retailer was not threatened which is important to allow sustainable implementation of online nudging as part of a public health prevention strategy. The adverse effects found on purchasing behaviors for those from non-deprived areas call for further research. Further research should also focus on real-life effects of online healthy food nudging as part of a broader nutrition intervention strategy, and on the equitability of the online nudging intervention within populations.

## Supplementary Information

**Additional file 1: Supplementary Figs. 1-5** and **Supplementary Tables 1-7**. **Supplementary Fig. 1**–Detailed description of the webpage banner explaining the nudging labels. **Supplementary Fig. 2**–Example of the webpage banner placement on the bread category webpage, combined with a tastiness label on whole-grain breads. **Supplementary Fig. 3**–Healthy product swaps (position nudge), including the tastiness labels and a heading ‘Also frequently purchased by other customers. **Supplementary Fig. 4**–Healthy check-out suggestions (position nudge), including the popularity labels and a heading ‘Tasty alternatives’. **Supplementary Fig. 5**–Flow diagram of shoppers. **Supplementary Table 1**–Type of information nudges and food group assignment 7. **Supplementary Table 2**–Mean percentages (95% CI) a of total healthy purchases and per food group in arm 1 (control arm), by area-level deprivation. **Supplementary Table 3**–Mean grams (95% CI) a of healthy and unhealthy purchases per food group in arm 1 (control arm), by area-level deprivation. **Supplementary Table 4**–Mean differences (95% CI) a in the percentage healthy purchases within food groups in Arm 2 (information nudge), Arm 3 (position nudge), and Arm 4 (information and position nudges) compared to arm 1, by area-level deprivation. **Supplementary Table 5**–Mean differences (95% CI) a in total retailer revenue (Euros) in Arm 2 (information nudge), Arm 3 (position nudge), and Arm 4 (information and position nudges) compared to arm 1, by area-level deprivation. **Supplementary Table 6**–Mean differences (95% CI) a in the grams healthy and grams unhealthy purchased within food groups in Arm 2 (information nudge), Arm 3 (position nudge), and Arm 4 (information and position nudges) compared to arm 1, by area-level deprivation. **Supplementary Table 7**–Mean differences (98% CI) a in the percentage healthy purchases within food groups in Arm 2 (information nudge), Arm 3 (position nudge), and Arm 4 (information and position nudges) compared to arm 1, by area-level deprivation. **Supplementary Table 8**–Mean differences (98% CI) a in the grams healthy and grams unhealthy purchased within food groups in Arm 2 (information nudge), Arm 3 (position nudge), and Arm 4 (information and position nudges) compared to arm 1, by area-level deprivation.

## Data Availability

The datasets analyzed during the current study are not publicly available as sharing of supermarket sales data will violate the data sharing agreement with the supermarket partner.

## Abbreviations

CI: Confidence interval
IP-addresses: Internet protocol addresses
IQR: Interquartile range
RCT: Randomized controlled trial
SD: Standard deviation
SEP: Socioeconomic position
TIPPME: Typology of interventions in proximal physical micro-environments
