# Supplementary material for: Nudging customers towards healthier food and beverage purchases in a real-life online supermarket: a multi-arm randomized controlled trial
Source: BMC Med. 2022 Jan 17;20:10. doi: 10.1186/s12916-021-02205-z (PMC8762859; doi:10.1186/s12916-021-02205-z)
Supplement: Supplementary file 1 — Additional file 1: Supplementary Figs. 1-5 and Supplementary Tables 1-7. Supplementary Fig. 1–Detailed description of the webpage banner explaining the nudging labels. Supplementary Fig. 2–Example of the webpage banner placement on the bread category webpage, combined with a tastiness label on whole-grain breads. Supplementary Fig. 3–Healthy product swaps (position nudge), including the tastiness labels and a heading ‘Also frequently purchased by other customers. Supplementary Fig. 4–Healthy check-out suggestions (position nudge), including the popularity labels and a heading ‘Tasty alternatives’. Supplementary Fig. 5–Flow diagram of shoppers. Supplementary Table 1–Type of information nudges and food group assignment 7. Supplementary Table 2–Mean percentages (95% CI) a of total healthy purchases and per food group in arm 1 (control arm), by area-level deprivation. Supplementary Table 3–Mean grams (95% CI) a of healthy and unhealthy purchases per food group in arm 1 (control arm), by area-level deprivation. Supplementary Table 4–Mean differences (95% CI) a in the percentage healthy purchases within food groups in Arm 2 (information nudge), Arm 3 (position nudge), and Arm 4 (information and position nudges) compared to arm 1, by area-level deprivation. Supplementary Table 5–Mean differences (95% CI) a in total retailer revenue (Euros) in Arm 2 (information nudge), Arm 3 (position nudge), and Arm 4 (information and position nudges) compared to arm 1, by area-level deprivation. Supplementary Table 6–Mean differences (95% CI) a in the grams healthy and grams unhealthy purchased within food groups in Arm 2 (information nudge), Arm 3 (position nudge), and Arm 4 (information and position nudges) compared to arm 1, by area-level deprivation. Supplementary Table 7–Mean differences (98% CI) a in the percentage healthy purchases within food groups in Arm 2 (information nudge), Arm 3 (position nudge), and Arm 4 (information and position nudges) compared to arm 1, by area-level d [file 12916_2021_2205_MOESM1_ESM.docx]

**Nudging customers towards healthier food and beverage purchases in a real-life online supermarket: A multi-arm randomized controlled trial**

Online supplementary material

*Josine M. Stuber et al*

[Supplementary Figure 1. Detailed description of the webpage banner explaining the nudging labels 2](#_Toc89100238)

[Supplementary Figure 2. Example of the webpage banner placement on the bread category webpage, combined with a tastiness label on whole-grain breads 3](#_Toc89100239)

[Supplementary Figure 3. Healthy product swaps (position nudge), including the tastiness labels and a heading *‘Also frequently purchased by other customers’* 4](#_Toc89100240)

[Supplementary Figure 4. Healthy check-out suggestions (position nudge), including the popularity labels and a heading ‘*Tasty alternatives*’ 5](#_Toc89100241)

[Supplementary Figure 5. Flow diagram of shoppers 6](#_Toc89100242)

[Supplementary Table 1. Type of information nudges and food group assignment 7](#_Toc89100243)

[Supplementary Table 2. Mean percentages (95% CI)^a^ of total healthy purchases and per food group in arm 1 (control arm), by area-level deprivation 8](#_Toc89100244)

[Supplementary Table 3. Mean grams (95% CI)^a^ of healthy and unhealthy purchases per food group in arm 1 (control arm), by area-level deprivation 9](#_Toc89100245)

[Supplementary Table 4. Mean differences (95% CI)^a^ in the percentage healthy purchases within food groups in Arm 2 (information nudge), Arm 3 (position nudge), and Arm 4 (information and position nudges) compared to arm 1, by area-level deprivation 10](#_Toc89100246)

[Supplementary Table 5. Mean differences (95% CI)^a^ in total retailer revenue (Euros) in Arm 2 (information nudge), Arm 3 (position nudge), and Arm 4 (information and position nudges) compared to arm 1, by area-level deprivation 12](#_Toc89100247)

[Supplementary Table 6. Mean differences (95% CI)^a^ in the grams healthy and grams unhealthy purchased within food groups in Arm 2 (information nudge), Arm 3 (position nudge), and Arm 4 (information and position nudges) compared to arm 1, by area-level deprivation 13](#_Toc89100248)

[Supplementary Table 7. Mean differences (98% CI)^a^ in the percentage healthy purchases within food groups in Arm 2 (information nudge), Arm 3 (position nudge), and Arm 4 (information and position nudges) compared to arm 1, by area-level deprivation 15](#_Toc89100249)

[Supplementary Table 8. Mean differences (98% CI)^a^ in the grams healthy and grams unhealthy purchased within food groups in Arm 2 (information nudge), Arm 3 (position nudge), and Arm 4 (information and position nudges) compared to arm 1, by area-level deprivation 17](#_Toc89100250)


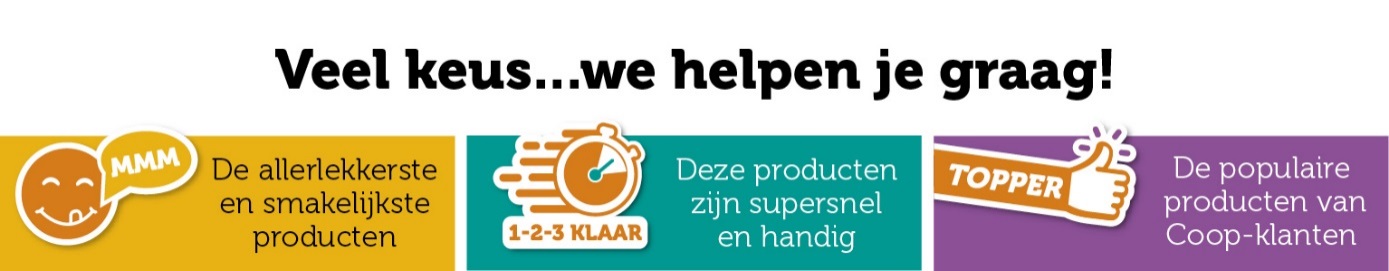


| ***‘Lots of choice…We are happy to help!’*** | | |
| --- | --- | --- |
| *‘Most delicious and*  *tastiest products’* | *‘Convenient and quickly*  *to prepare products’* | *‘Most popular products*  *from Coop customers’* |
|  |  |  |
|  |  |  |

# Supplementary Figure 1. Detailed description of the webpage banner explaining the nudging labels

***
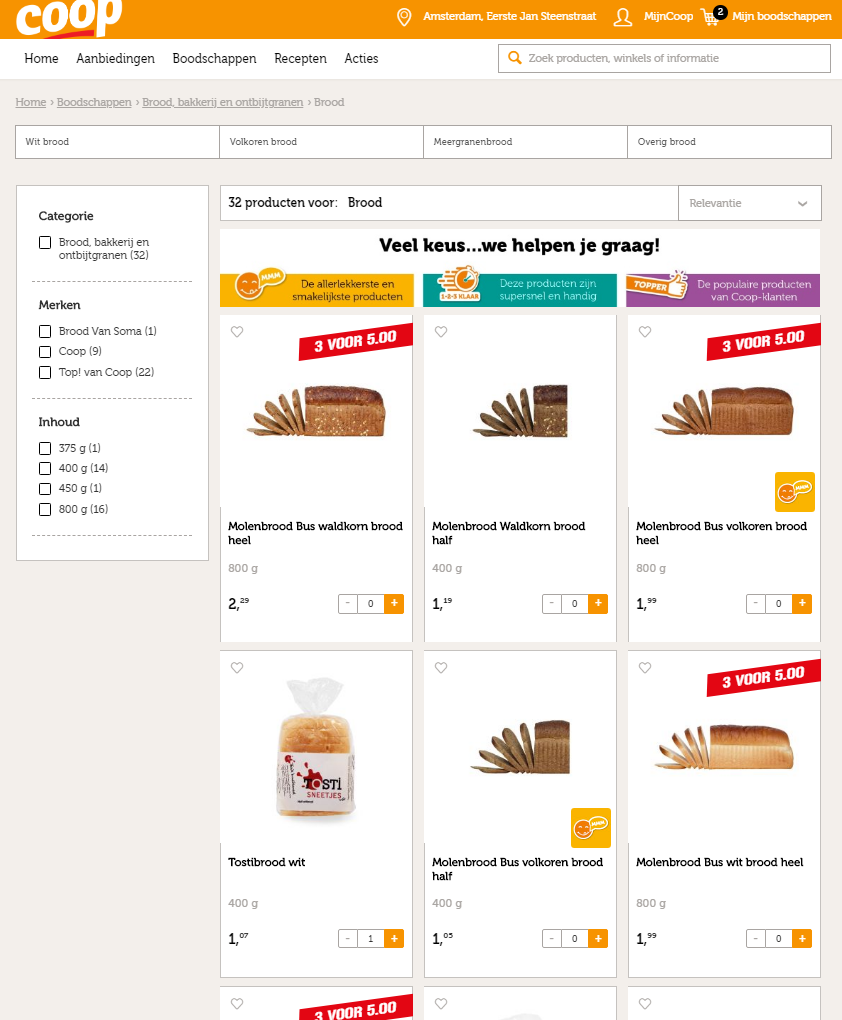
***

# Supplementary Figure 2. Example of the webpage banner placement on the bread category webpage, combined with a tastiness label on whole-grain breads

**
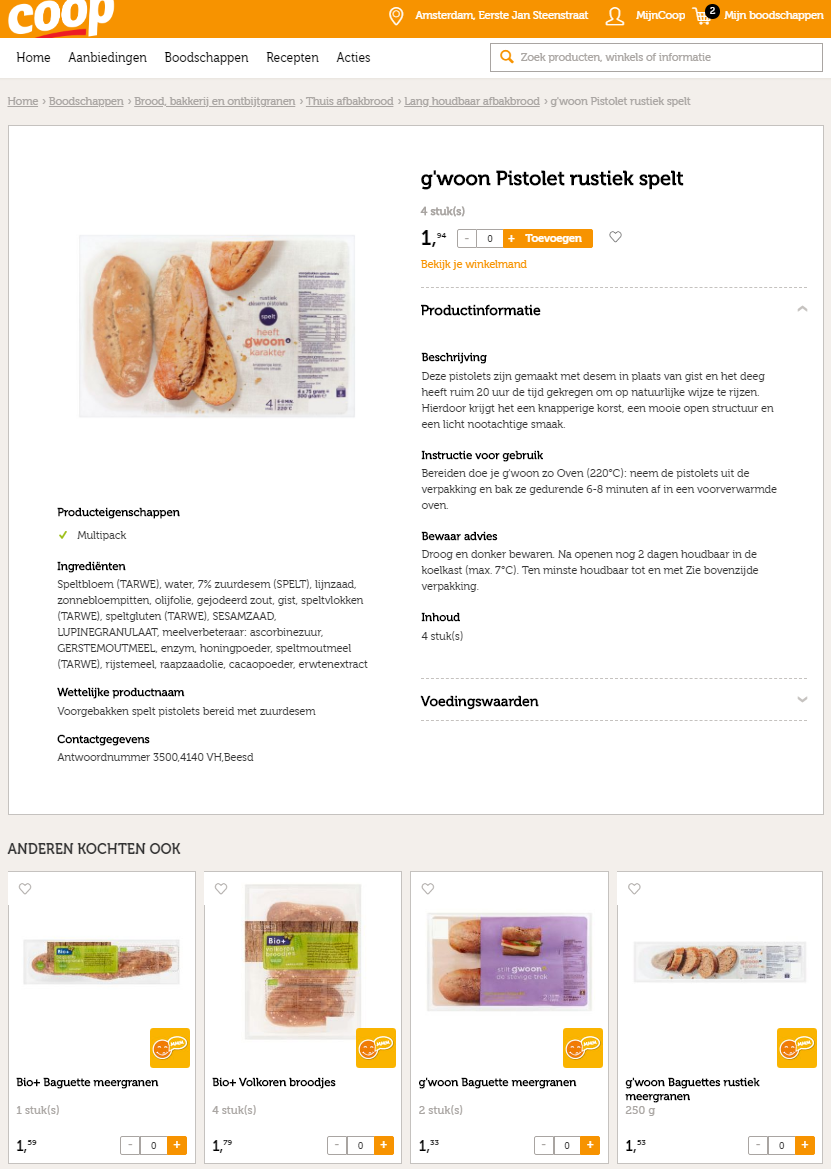
**

# Supplementary Figure 3. Healthy product swaps (position nudge), including the tastiness labels and a heading *‘Also frequently purchased by other customers’*

**
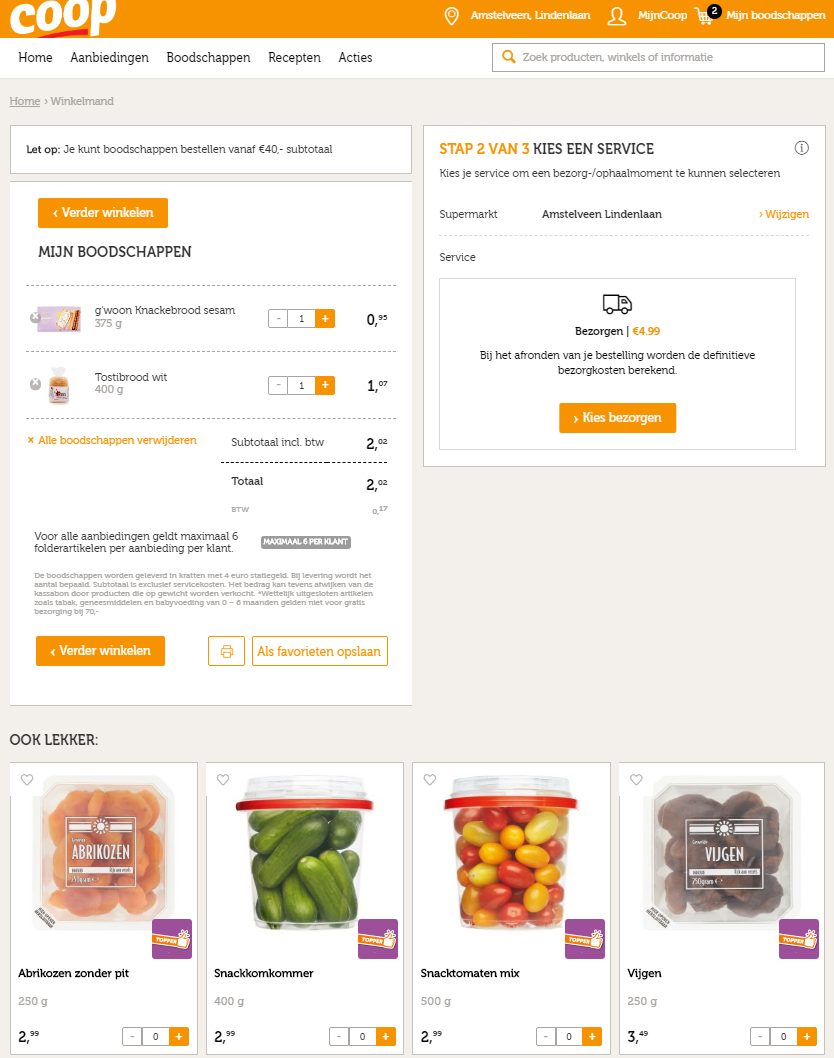
**

# Supplementary Figure 4. Healthy check-out suggestions (position nudge), including the popularity labels and a heading ‘*Tasty alternatives*’

# Supplementary Figure 5. Flow diagram of shoppers

#

# Supplementary Table 1. Type of information nudges and food group assignment

| **Tastiness label** | **Convenience label** | **Popularity label** |
| --- | --- | --- |
| - Breads and bread substitutes - Breakfast grains - Pasta and rice - Yogurt products - Cheeses - Peanut butters | - Canned legumes - Canned fish - Pre-cut and frozen vegetables - Pre-cut and frozen fruits - Nuts | - Fresh vegetables - Fresh and dried fruits - Cooking fats and butters - Fresh fish - Milk products - Teas and coffees - Sodas and waters |

# Supplementary Table 2. Mean percentages (95% CI)^a^ of total healthy purchases and per food group in arm 1 (control arm), by area-level deprivation

|  | **Deprived areas** | | | **Non-deprived areas** | | |
| --- | --- | --- | --- | --- | --- | --- |
|  | **Mean** | **SD** |  | **Mean** | **SD** |  |
| **Total healthy purchases** | 36.1 | 22.2 |  | 40.1 | 21.9 |  |
|  | **Mean** | **95%** | **CI** | **Mean** | **95%** | **CI** |
| **Fruits** | 89.4 | 87.6, | 91.1 | 90.3 | 88.0, | 92.4 |
| **Vegetables** | 85.7 | 83.7, | 87.6 | 89.0 | 87.5, | 90.5 |
| **Breads** | 26.4 | 22.8, | 30.2 | 26.2 | 22.1, | 30.5 |
| **Bread substitutes** | 18.0 | 14.0, | 22.1 | 20.5 | 16.8, | 24.4 |
| **Potatoes** | 70.9 | 67.0, | 75.0 | 74.1 | 70.2, | 77.7 |
| **Pasta and rice** | 13.6 | 9.9, | 17.5 | 19.6 | 15.2, | 24.1 |
| **Teas and coffees** | 86.9 | 82.7, | 90.7 | 92.3 | 89.0, | 95.1 |
| **Sodas, juices and waters** | 11.4 | 8.8, | 14.1 | 12.7 | 10.1, | 15.5 |
| **Cheeses** | 12.7 | 10.2, | 15.4 | 10.9 | 8.6, | 13.2 |
| **Milk and yogurt products** | 48.1 | 45.4, | 50.9 | 54.0 | 50.8, | 57.1 |
| **Meats** | 34.5 | 31.5, | 37.7 | 35.4 | 32.3, | 38.4 |
| **Fish** | 65.5 | 60.2, | 70.5 | 75.4 | 70.4, | 80.1 |
| **Legumes** | 42.7 | 36.1, | 49.2 | 44.1 | 37.1, | 51.0 |
| **Nuts** | 29.8 | 23.9, | 35.9 | 34.4 | 28.0, | 40.8 |
| **Fats** | 46.2 | 41.8, | 50.6 | 46.4 | 42.7, | 50.1 |
| **Other foods^b^** | 10.7 | 9.9, | 11.5 | 9.3 | 8.7, | 10.0 |
| **Savoury snacks^b^** | 4.6 | 4.2, | 5.0 | 4.0 | 3.7, | 4.5 |
| **Sweet snacks^b^** | 6.6 | 6.2, | 7.1 | 6.0 | 5.6, | 6.4 |
| **Alcoholic drinks^c^** | 29.7 | 26.5, | 32.9 | 26.3 | 23.9, | 28.8 |

The number of shoppers within each food group is presented in Table 2.

^a^ Based on 10,000 non-parametric bootstrap replicates

^b^ Percentage was calculated based on the total grams purchased (considering the lack of healthy products in these food groups) excluding the grams purchased from alcohol

^c^ Percentage was calculated based on the total grams purchased

# Supplementary Table 3. Mean grams (95% CI)^a^ of healthy and unhealthy purchases per food group in arm 1 (control arm), by area-level deprivation

|  | **Grams Healthy** | | | | | | **Grams unhealthy** | | | | | |
| --- | --- | --- | --- | --- | --- | --- | --- | --- | --- | --- | --- | --- |
|  | **Deprived areas** | | | **Non-deprived areas** | | | **Deprived areas** | | | **Non-deprived areas** | | |
|  | **Mean** | **95% CI** | | **Mean** | **95% CI** | | **Mean** | **95% CI** | | **Mean** | **95% CI** | |
| **Fruits** | 1620 | 1458, | 1811 | 1997 | 1801, | 2217 | 1039 | 910, | 1176 | 1245 | 1025, | 1475 |
| **Vegetables** | 1968 | 1813, | 2134 | 2460 | 2255, | 2683 | 953 | 858, | 1058 | 803 | 720, | 891 |
| **Breads** | 1459 | 1301, | 1631 | 1386 | 1176, | 1661 | 1493 | 1384, | 1606 | 1759 | 1577, | 1956 |
| **Bread substitutes** | 136 | 102, | 171 | 202 | 148, | 265 | 582 | 528, | 640 | 610 | 551, | 673 |
| **Potatoes** | 1249 | 1083, | 1439 | 1245 | 1119, | 1381 | 835 | 749, | 929 | 854 | 761, | 958 |
| **Pasta and rice** | 850 | 698, | 1031 | 770 | 682, | 858 | 1123 | 956, | 1337 | 914 | 829, | 1009 |
| **Teas and coffees** | 585 | 485, | 702 | 555 | 492, | 622 | 271 | 192, | 365 | 516 | 281, | 809 |
| **Sodas, juices and waters** | 18482 | 14229, | 23037 | 12461 | 9982, | 15045 | 7571 | 6809, | 8421 | 7596 | 6802, | 8474 |
| **Cheeses** | 276 | 246, | 306 | 259 | 236, | 284 | 652 | 591, | 725 | 651 | 599, | 706 |
| **Milk and yogurt products** | 4574 | 4079, | 5151 | 5254 | 4716, | 5838 | 3195 | 2930, | 3472 | 3400 | 3048, | 3775 |
| **Meats** | 883 | 827, | 942 | 966 | 893, | 1045 | 979 | 896, | 1067 | 976 | 907, | 1049 |
| **Fish** | 469 | 420, | 523 | 476 | 420, | 535 | 548 | 492, | 608 | 574 | 473, | 714 |
| **Legumes** | 838 | 690, | 999 | 849 | 720, | 1005 | 984 | 825, | 1167 | 888 | 751, | 1035 |
| **Nuts** | 354 | 302, | 412 | 332 | 290, | 372 | 476 | 436, | 520 | 563 | 497, | 650 |
| **Fats** | 890 | 804, | 986 | 905 | 801, | 1029 | 719 | 667, | 773 | 773 | 693, | 857 |
| **Other foods** | **NA** |  | | **NA** |  | | 1971 | 1835, | 2122 | 2006 | 1878, | 2144 |
| **Savoury snacks** | **NA** |  | | **NA** |  | | 853 | 778, | 933 | 846 | 772, | 928 |
| **Sweet snacks** | **NA** |  | | **NA** |  | | 1251 | 1170, | 1333 | 1274 | 1164, | 1396 |
| **Alcoholic drinks** | **NA** |  | | **NA** |  | | 8545 | 7477, | 9705 | 7897 | 6736, | 9211 |

The number of shoppers within each food group is presented in Table 2.

NA: Not applicable.

^a^ Based on 10,000 non-parametric bootstrap replicates.

# Supplementary Table 4. Mean differences (95% CI)^a^ in the percentage healthy purchases within food groups in Arm 2 (information nudge), Arm 3 (position nudge), and Arm 4 (information and position nudges) compared to arm 1, by area-level deprivation

|  | **Deprived areas** | | | **Non-deprived areas** | | |
| --- | --- | --- | --- | --- | --- | --- |
|  | **Mean diff.** | **95%** | **CI** | **Mean diff.** | **95%** | **CI** |
| **Fruits** |  |  |  |  |  |  |
| *Arm 2* | 0.9 | -1.8, | 3.5 | -1.3 | -4.1, | 1.5 |
| *Arm 3* | 1.2 | -1.3, | 3.6 | -1.3 | -4.0, | 1.3 |
| *Arm 4* | -0.7 | -3.7, | 2.3 | **-3.0** | **-5.8,** | **-0.2** |
| **Vegetables** |  |  |  |  |  |  |
| *Arm 2* | -0.6 | -3.6, | 2.3 | -2.1 | -4.5, | 0.2 |
| *Arm 3* | 0.1 | -2.5, | 2.8 | -2.1 | -4.6, | 0.4 |
| *Arm 4* | -0.1 | -3.2, | 2.9 | **-2.8** | **-5.5,** | **-0.3** |
| **Breads** |  |  |  |  |  |  |
| *Arm 2* | 5.0 | -0.3, | 10.1 | 4.7 | -1.0, | 10.5 |
| *Arm 3* | 1.1 | -3.6, | 5.9 | 3.5 | -1.5, | 8.4 |
| *Arm 4* | 3.5 | -1.6, | 8.3 | 3.3 | -2.2, | 8.4 |
| **Bread substitutes** | |  |  |  |  |  |
| *Arm 2* | 0.5 | -4.9, | 6.0 | -1.4 | -7.2, | 4.5 |
| *Arm 3* | 1.8 | -4.5, | 8.2 | 0.4 | -5.0, | 5.9 |
| *Arm 4* | 3.2 | -2.6, | 9.2 | -3.5 | -8.6, | 1.8 |
| **Potatoes** |  |  |  |  |  |  |
| *Arm 2* | -1.9 | -7.9, | 4.0 | 3.5 | -1.8, | 8.7 |
| *Arm 3* | -0.5 | -6.6, | 5.5 | 2.6 | -2.9, | 8.0 |
| *Arm 4* | 1.4 | -4.2, | 7.0 | -1.0 | -7.3, | 5.4 |
| **Pasta and rice** |  |  |  |  |  |  |
| *Arm 2* | 4.1 | -1.4, | 9.6 | -2.3 | -8.3, | 3.9 |
| *Arm 3* | 2.6 | -4.1, | 9.5 | -1.2 | -7.5, | 5.7 |
| *Arm 4* | **7.6** | **2.4,** | **12.7** | -0.4 | -5.8, | 5.4 |
| **Teas and coffees** |  |  |  |  |  |  |
| *Arm 2* | 1.1 | -4.2, | 6.6 | -4.2 | -9.4, | 1.0 |
| *Arm 3* | 0.0 | -5.0, | 5.2 | -1.5 | -5.8, | 2.7 |
| *Arm 4* | 2.1 | -3.0, | 7.4 | 1.7 | -1.9, | 5.6 |
| **Sodas, waters and juices** |  |  |  |  |  |  |
| *Arm 2* | 1.6 | -2.3, | 5.6 | -1.3 | -5.2, | 2.7 |
| *Arm 3* | -0.5 | -4.2, | 3.4 | 0.1 | -3.4, | 3.7 |
| *Arm 4* | -0.7 | -3.9, | 2.6 | -0.5 | -3.9, | 2.9 |
| **Cheeses** |  |  |  |  |  |  |
| *Arm 2* | 0.4 | -3.3, | 4.1 | -0.9 | -3.6, | 1.9 |
| *Arm 3* | -2.9 | -6.2, | 0.4 | 1.0 | -2.3, | 4.6 |
| *Arm 4* | -2.8 | -6.1, | 0.6 | -0.2 | -3.2, | 2.9 |
| **Milk and yogurt products** |  |  |  |  |  |  |
| *Arm 2* | **4.6** | **0.5,** | **8.8** | -1.2 | -5.2, | 2.8 |
| *Arm 3* | 0.0 | -3.9, | 3.9 | -0.0 | -3.8, | 3.7 |
| *Arm 4* | 4.2 | -0.1, | 8.6 | -0.5 | -5.0, | 3.8 |
| **Meats** |  |  |  |  |  |  |
| *Arm 2* | -0.1 | -3.8, | 3.5 | 0.2 | -3.3, | 3.5 |
| *Arm 3* | 0.6 | -3.6, | 4.8 | 0.0 | -3.6, | 3.7 |
| *Arm 4* | -0.8 | -4.5, | 2.8 | -2.9 | -6.4, | 0.6 |
| **Fish** |  |  |  |  |  |  |
| *Arm 2* | 7.1 | -0.5, | 14.8 | **-8.4** | **-16.5,** | **-0.0** |
| *Arm 3* | 5.7 | -1.2, | 12.5 | -3.5 | -11.1, | 4.3 |
| *Arm 4* | 5.3 | -2.4, | 12.8 | -2.1 | -9.8, | 5.5 |
| **Legumes** |  |  |  |  |  |  |
| *Arm 2* | 0.6 | -7.9, | 9.3 | **-10.2** | **-19.3,** | **-1.0** |
| *Arm 3* | -0.4 | -10.3, | 9.8 | -2.2 | -11.6, | 7.2 |
| *Arm 4* | -5.5 | -16.1, | 5.0 | 5.6 | -3.6, | 14.8 |
| **Nuts** |  |  |  |  |  |  |
| *Arm 2* | 1.0 | -7.7, | 9.8 | **-10.7** | **-17.5,** | **-3.8** |
| *Arm 3* | -4.7 | -11.9, | 2.4 | -4.6 | -12.5, | 3.6 |
| *Arm 4* | -2.7 | -10.7, | 5.4 | -3.8 | -12.2, | 4.4 |
| **Fats** |  |  |  |  |  |  |
| *Arm 2* | 1.6 | -4.4, | 7.6 | **-7.9** | **-13.1,** | **-2.7** |
| *Arm 3* | 1.7 | -4.1, | 7.5 | -5.6 | -11.0, | 0.0 |
| *Arm 4* | -2.9 | -8.3, | 2.6 | -2.7 | -8.0, | 2.5 |
| **Unhealthy other foods^b^** |  |  |  |  |  |  |
| *Arm 2* | **-1.1** | **-2.2,** | **-0.0** | 0.1 | -0.7, | 1.0 |
| *Arm 3* | -0.5 | -1.4, | 0.4 | -0.0 | -0.9, | 0.8 |
| *Arm 4* | -0.1 | -1.3, | 1.2 | 0.1 | -0.9, | 1.0 |
| **Unhealthy savoury snacks^b^** |  |  |  |  |  |  |
| *Arm 2* | -0.6 | -1.1, | 0.0 | -0.0 | -0.6, | 0.6 |
| *Arm 3* | -0.3 | -0.9, | 0.4 | 0.1 | -0.4, | 0.7 |
| *Arm 4* | -0.1 | -0.8, | 0.5 | -0.3 | -0.8, | 0.2 |
| **Unhealthy sweet snacks^b^** |  |  |  |  |  |  |
| *Arm 2* | -0.4 | -0.9, | 0.2 | 0.2 | -0.4, | 0.8 |
| *Arm 3* | -0.1 | -0.8, | 0.6 | 0.1 | -0.5, | 0.7 |
| *Arm 4* | -0.5 | -1.1, | 0.2 | 0.3 | -0.3, | 0.9 |
| **Unhealthy alcohol drinks^c^** |  |  |  |  |  |  |
| *Arm 2* | -2.8 | -7.2, | 1.6 | -0.1 | -3.7, | 3.4 |
| *Arm 3* | -2.9 | -7.1, | 1.5 | -0.1 | -3.7, | 3.2 |
| *Arm 4* | -0.5 | -5.1, | 4.2 | -0.5 | -3.8, | 2.9 |

The number of shoppers within each food group is presented in Table 2.

^a^ Based on 10,000 non-parametric bootstrap replicates; Bold numbers represent statistically significant results.

^b^ Percentage difference was not based on the grams of healthy products purchased, but calculated based on the total grams purchased (considering the lack of healthy products in these food groups) excluding the grams purchased from alcohol

^c^ Percentage difference was not based on the grams of healthy products purchased, but calculated based on the total grams purchased (considering the lack of healthy products in these food groups)

# Supplementary Table 5. Mean differences (95% CI)^a^ in total retailer revenue (Euros) in Arm 2 (information nudge), Arm 3 (position nudge), and Arm 4 (information and position nudges) compared to arm 1, by area-level deprivation

|  | **Deprived areas** | | | **Non-deprived areas** | | |
| --- | --- | --- | --- | --- | --- | --- |
|  | **Mean diff.** | **95%** | **CI** | **Mean diff.** | **95%** | **CI** |
| *Arm 2* | 2.06 | -2.67; | 6.94 | -1.88 | -6.50; | 2.92 |
| *Arm 3* | 3.17 | -1.57; | 8.30 | -1.97 | -6.91; | 2.90 |
| *Arm 4* | 1.71 | -2.46; | 5.86 | 0.74 | -4.07; | 5.67 |

The number of shoppers within each group is presented in Table 2.

^a^ Based on 10,000 non-parametric bootstrap replicates

# Supplementary Table 6. Mean differences (95% CI)^a^ in the grams healthy and grams unhealthy purchased within food groups in Arm 2 (information nudge), Arm 3 (position nudge), and Arm 4 (information and position nudges) compared to arm 1, by area-level deprivation

|  | **Grams healthy** | | | | | | **Grams unhealthy** | | | | | |
| --- | --- | --- | --- | --- | --- | --- | --- | --- | --- | --- | --- | --- |
|  | **Deprived areas** | | | **Non-deprived areas** | | | **Deprived areas** | | | **Non-deprived areas** | | |
|  | **Mean diff.** | **95%** | **CI** | **Mean diff.** | **95%** | **CI** | **Mean diff.** | **95%** | **CI** | **Mean diff.** | **95%** | **CI** |
| **Fruits** |  |  |  |  |  |  |  |  |  |  |  |  |
| *Arm 2* | 126 | -174, | 462 | **-366** | **-639,** | **-119** | 59 | -170, | 304 | -129 | -390, | 127 |
| *Arm 3* | -79 | -299, | 116 | **-331** | **-582,** | **-91** | 60 | -165, | 314 | -48 | -322, | 237 |
| *Arm 4* | -14 | -253, | 227 | **-352** | **-599,** | **-125** | -8 | -196, | 183 | 53 | -231, | 334 |
| **Vegetables** |  |  |  |  |  |  |  |  |  |  |  |  |
| *Arm 2* | **271** | **15,** | **560** | -263 | -533, | 3 | 20 | -133, | 190 | -37 | -149, | 77 |
| *Arm 3* | 119 | -93, | 334 | -212 | -478, | 38 | -108 | -244, | 23 | 35 | -76, | 144 |
| *Arm 4* | **249** | **41,** | **452** | -122 | -397, | 145 | -13 | -160, | 138 | 85 | -43, | 223 |
| **Breads** |  |  |  |  |  |  |  |  |  |  |  |  |
| *Arm 2* | 40 | -227, | 319 | -224 | -526, | 23 | 38 | -110, | 181 | -148 | -393, | 103 |
| *Arm 3* | -41 | -284, | 190 | 74 | -232, | 347 | 105 | -49, | 259 | -145 | -384, | 121 |
| *Arm 4* | -39 | -258, | 183 | -15 | -298, | 235 | 168 | -32, | 367 | -163 | -397, | 62 |
| **Bread substitutes** | |  |  |  |  |  |  |  |  |  |  |  |
| *Arm 2* | 1 | -48, | 50 | -40 | -115, | 27 | 25 | -54, | 105 | -2 | -95, | 99 |
| *Arm 3* | 7 | -42, | 57 | -55 | -125, | 7 | 38 | -45, | 129 | -25 | -110, | 58 |
| *Arm 4* | 29 | -25, | 84 | -58 | -133, | 7 | -24 | -109, | 66 | 76 | -62, | 246 |
| **Potatoes** |  |  |  |  |  |  |  |  |  |  |  |  |
| *Arm 2* | 219 | -50, | 486 | 144 | -91, | 384 | 63 | -100, | 238 | 49 | -86, | 183 |
| *Arm 3* | 9 | -240, | 249 | 158 | -53, | 376 | -4 | -120, | 111 | 49 | -99, | 193 |
| *Arm 4* | 8 | -248, | 242 | 108 | -100, | 316 | 50 | -93, | 212 | 107 | -81, | 312 |
|  |  |  |  |  |  |  |  |  |  |  |  |  |
| **Pasta and rice** |  |  |  |  |  |  |  |  |  |  |  |  |
| *Arm 2* | 11 | -242, | 283 | 121 | -15, | 267 | -68 | -313, | 147 | 2 | -118, | 124 |
| *Arm 3* | 87 | -188, | 339 | -24 | -146, | 101 | **-231** | **-461,** | **-38** | -13 | -148, | 127 |
| *Arm 4* | -1 | -244, | 231 | 63 | -64, | 208 | **-274** | **-505,** | **-75** | -52 | -165, | 58 |
| **Teas and coffees** |  |  |  |  |  |  |  |  |  |  |  |  |
| *Arm 2* | -7 | -150, | 142 | 1 | -71, | 75 | 44 | -44, | 132 | **-246** | **-540,** | **-6** |
| *Arm 3* | -15 | -170, | 136 | 6 | -85, | 95 | 54 | -61, | 166 | -206 | -511, | 51 |
| *Arm 4* | -34 | -171, | 89 | 31 | -57, | 128 | 2 | -122, | 127 | **-273** | **-576,** | **-21** |
| **Sodas, waters and juices** |  |  |  |  |  |  |  |  |  |  |  |  |
| *Arm 2* | -1209 | -7893, | 5790 | **15051** | **1618,** | **37328** | -318 | -1465, | 814 | 154 | -1031, | 1315 |
| *Arm 3* | **-6078** | **-11643,** | **-579** | 4604 | -329, | 10649 | 786 | -273, | 1933 | -650 | -1654, | 281 |
| *Arm 4* | -1830 | -8028, | 4759 | -243 | -4183, | 3877 | -398 | -1468, | 624 | -664 | -1852, | 508 |
| **Cheeses** |  |  |  |  |  |  |  |  |  |  |  |  |
| *Arm 2* | 35 | -14, | 88 | 6 | -42, | 64 | -23 | -114, | 70 | **-76** | **-140,** | **-13** |
| *Arm 3* | 13 | -39, | 74 | **55** | **15,** | **98** | -14 | -97, | 60 | -5 | -82, | 74 |
| *Arm 4* | 45 | -19, | 119 | **51** | **11,** | **91** | -6 | -98, | 83 | 45 | -32, | 128 |
| **Milk and yogurt products** |  |  |  |  |  |  |  |  |  |  |  |  |
| *Arm 2* | 580 | -457, | 1793 | -100 | -642, | 509 | 322 | -282, | 1112 | -75 | -526, | 372 |
| *Arm 3* | 119 | -654, | 904 | -138 | -771, | 539 | 138 | -307, | 644 | 35 | -403, | 468 |
| *Arm 4* | 139 | -599, | 879 | -183 | -813, | 466 | -61 | -441, | 300 | 47 | -368, | 469 |
| **Meats** |  |  |  |  |  |  |  |  |  |  |  |  |
| *Arm 2* | 92 | -12, | 203 | -43 | -142, | 55 | 22 | -113, | 173 | 18 | -78, | 110 |
| *Arm 3* | 74 | -22, | 184 | -21 | -111, | 65 | 9 | -96, | 112 | 54 | -43, | 149 |
| *Arm 4* | 36 | -44, | 116 | -45 | -143, | 49 | 97 | -24, | 219 | **128** | **23,** | **238** |
| **Fish** |  |  |  |  |  |  |  |  |  |  |  |  |
| *Arm 2* | 49 | -63, | 171 | -63 | -135, | 10 | 101 | -44, | 266 | 3 | -165, | 167 |
| *Arm 3* | 8 | -56, | 72 | -24 | -97, | 47 | -29 | -110, | 52 | -71 | -221, | 48 |
| *Arm 4* | 62 | -33, | 164 | 14 | -66, | 100 | -18 | -110, | 71 | 47 | -112, | 224 |
| **Legumes** |  |  |  |  |  |  |  |  |  |  |  |  |
| *Arm 2* | 39 | -177, | 261 | -87 | -269, | 92 | -58 | -267, | 141 | -37 | -193, | 108 |
| *Arm 3* | -22 | -214, | 167 | -96 | -286, | 81 | -152 | -361, | 49 | 42 | -153, | 240 |
| *Arm 4* | 8 | -205, | 211 | 5 | -224, | 236 | -50 | -295, | 202 | -31 | -211, | 149 |
| **Nuts** |  |  |  |  |  |  |  |  |  |  |  |  |
| *Arm 2* | 12 | -74, | 99 | -23 | -76, | 32 | 35 | -28, | 100 | -28 | -125, | 54 |
| *Arm 3* | 16 | -74, | 114 | -7 | -68, | 59 | **79** | **4,** | **159** | -8 | -97, | 70 |
| *Arm 4* | -19 | -84, | 43 | 24 | -49, | 108 | 65 | -4, | 139 | -30 | -124, | 53 |
| **Fats** |  |  |  |  |  |  |  |  |  |  |  |  |
| *Arm 2* | 29 | -97, | 153 | -88 | -243, | 51 | 51 | -43, | 146 | 23 | -79, | 124 |
| *Arm 3* | -22 | -138, | 89 | -17 | -191, | 155 | 5 | -76, | 85 | 3 | -84, | 89 |
| *Arm 4* | -18 | -144, | 104 | -26 | -165, | 100 | -61 | -143, | 22 | -2 | -107, | 107 |
| **Other foods** |  |  |  |  |  |  |  |  |  |  |  |  |
| *Arm 2* | NA |  |  | NA |  |  | -50 | -259, | 152 | -160 | -337, | 12 |
| *Arm 3* | NA |  |  | NA |  |  | -74 | -266, | 118 | **-196** | **-346,** | **-50** |
| *Arm 4* | NA |  |  | NA |  |  | -75 | -264, | 109 | -154 | -342, | 49 |
| **Savoury snacks** |  |  |  |  |  |  |  |  |  |  |  |  |
| *Arm 2* | NA |  |  | NA |  |  | -24 | -142, | 91 | 43 | -76, | 163 |
| *Arm 3* | NA |  |  | NA |  |  | -66 | -153, | 22 | -14 | -118, | 90 |
| *Arm 4* | NA |  |  | NA |  |  | -32 | -140, | 75 | 12 | -100, | 125 |
| **Sweet snacks** |  |  |  |  |  |  |  |  |  |  |  |  |
| *Arm 2* | NA |  |  | NA |  |  | -11 | -127, | 109 | -43 | -155, | 66 |
| *Arm 3* | NA |  |  | NA |  |  | 64 | -147, | 384 | -90 | -204, | 22 |
| *Arm 4* | NA |  |  | NA |  |  | -78 | -190, | 32 | 23 | -128, | 181 |
| **Alcoholic drinks** |  |  |  |  |  |  |  |  |  |  |  |  |
| *Arm 2* | NA |  |  | NA |  |  | 72 | -2025, | 2465 | 641 | -1329, | 2675 |
| *Arm 3* | NA |  |  | NA |  |  | -142 | -1879, | 1571 | 573 | -1193, | 2151 |
| *Arm 4* | NA |  |  | NA |  |  | 2477 | -370, | 5761 | 710 | -1065, | 2453 |

The number of shoppers within each food group is presented in Table 2; NA: Not applicable.

^a^ Based on 10,000 non-parametric bootstrap replicates; Bold numbers represent statistically significant results.

# Supplementary Table 7. Mean differences (98% CI)^a^ in the percentage healthy purchases within food groups in Arm 2 (information nudge), Arm 3 (position nudge), and Arm 4 (information and position nudges) compared to arm 1, by area-level deprivation

|  | **Deprived areas** | | | **Non-deprived areas** | | |
| --- | --- | --- | --- | --- | --- | --- |
|  | **Mean diff.** | **98%** | **CI** | **Mean diff.** | **98%** | **CI** |
| **Fruits** |  |  |  |  |  |  |
| *Arm 2* | 0.9 | -2.2, | 3.9 | -1.3 | -4.8, | 2.1 |
| *Arm 3* | 1.2 | -1.8, | 4.0 | -1.3 | -4.5, | 1.7 |
| *Arm 4* | -0.7 | -4.2, | 2.9 | -3.1 | -6.6, | 0.2 |
| **Vegetables** |  |  |  |  |  |  |
| *Arm 2* | -0.6 | -4.2, | 3.0 | -2.1 | -4.9, | 0.7 |
| *Arm 3* | 0.1 | -2.9, | 3.2 | -2.1 | -5.1, | 0.8 |
| *Arm 4* | -0.1 | -3.7, | 3.6 | -2.8 | -6.0, | 0.2 |
| **Breads** |  |  |  |  |  |  |
| *Arm 2* | 5.0 | -1.2, | 11.0 | 4.7 | -2.0, | 11.6 |
| *Arm 3* | 1.1 | -4.4, | 6.7 | 3.5 | -2.5, | 9.5 |
| *Arm 4* | 3.5 | -2.5, | 9.2 | 3.3 | -3.3, | 9.3 |
| **Bread substitutes** | |  |  |  |  |  |
| *Arm 2* | 0.4 | -6.0, | 7.0 | -1.3 | -8.2, | 5.5 |
| *Arm 3* | 1.8 | -5.7, | 9.5 | 0.5 | -5.9, | 7.3 |
| *Arm 4* | 3.1 | -3.7, | 10.4 | -3.5 | -9.6, | 2.7 |
| **Potatoes** |  |  |  |  |  |  |
| *Arm 2* | -1.9 | -9.0, | 5.0 | 3.4 | -2.7, | 9.7 |
| *Arm 3* | -0.4 | -7.4, | 6.8 | 2.6 | -3.8, | 9.0 |
| *Arm 4* | 1.4 | -5.2, | 8.1 | -1.0 | -8.3, | 6.6 |
| **Pasta and rice** |  |  |  |  |  |  |
| *Arm 2* | 4.1 | -2.4, | 10.7 | -2.3 | -9.3, | 5.0 |
| *Arm 3* | 2.6 | -5.5, | 10.6 | -1.2 | -8.7, | 7.1 |
| *Arm 4* | **7.6** | **1.1,** | **13.6** | -0.4 | -6.7, | 6.7 |
| **Teas and coffees** |  |  |  |  |  |  |
| *Arm 2* | 1.1 | -5.2, | 7.6 | -4.2 | -10.3, | 2.0 |
| *Arm 3* | 0.1 | -6.0, | 6.3 | -1.4 | -6.5, | 3.7 |
| *Arm 4* | 2.0 | -3.8, | 8.6 | 1.7 | -2.5, | 6.3 |
| **Sodas, waters and juices** |  |  |  |  |  |  |
| *Arm 2* | 1.6 | -3.1, | 6.3 | -1.3 | -6.0, | 3.3 |
| *Arm 3* | -0.5 | -4.6, | 4.2 | 0.1 | -4.0, | 4.4 |
| *Arm 4* | -0.6 | -4.5, | 3.2 | -0.5 | -4.6, | 3.4 |
| **Cheeses** |  |  |  |  |  |  |
| *Arm 2* | 0.4 | -3.93, | 4.7 | -0.9 | -4.0, | 2.4 |
| *Arm 3* | -3.0 | -6.9, | 1.1 | 1.0 | -3.0, | 5.3 |
| *Arm 4* | -2.8 | -6.8, | 1.2 | -0.3 | -3.8, | 3.4 |
| **Milk and yogurt products** |  |  |  |  |  |  |
| *Arm 2* | 4.6 | -0.3, | 9.7 | -1.2 | -6.0, | 3.7 |
| *Arm 3* | 0.0 | -4.6, | 4.6 | 0.0 | -4.5, | 4.5 |
| *Arm 4* | 4.2 | -0.8, | 9.5 | -0.5 | -5.7, | 4.8 |
| **Meats** |  |  |  |  |  |  |
| *Arm 2* | -0.1 | -4.6, | 4.2 | 0.1 | -3.9, | 4.2 |
| *Arm 3* | 0.6 | -4.2, | 5.5 | 0.0 | -4.3, | 4.4 |
| *Arm 4* | -0.8 | -5.2, | 3.4 | -2.8 | -7.1, | 1.4 |
| **Fish** |  |  |  |  |  |  |
| *Arm 2* | 7.2 | -1.7, | 16.2 | -8.4 | -18.0, | 1.2 |
| *Arm 3* | 5.8 | -2.1, | 13.8 | -3.5 | -12.7, | 5.9 |
| *Arm 4* | 5.3 | -3.8, | 14.5 | -2.1 | -10.8, | 7.0 |
| **Legumes** |  |  |  |  |  |  |
| *Arm 2* | 0.6 | -9.8, | 10.7 | -10.1 | -20.9, | 0.9 |
| *Arm 3* | -0.4 | -12.1, | 11.6 | -2.1 | -13.1, | 8.9 |
| *Arm 4* | -5.6 | -17.9, | 6.8 | 5.7 | -4.9, | 16.4 |
| **Nuts** |  |  |  |  |  |  |
| *Arm 2* | 1.0 | -9.4, | 11.4 | **-10.7** | **-19.0,** | **-2.2** |
| *Arm 3* | -4.6 | -13.6, | 4.1 | -4.6 | -14.3, | 5.1 |
| *Arm 4* | -2.6 | -12.6, | 7.4 | -3.8 | -14.1, | 6.0 |
| **Fats** |  |  |  |  |  |  |
| *Arm 2* | 1.5 | -5.7, | 8.8 | **-7.9** | **-14.5,** | **-1.7** |
| *Arm 3* | 1.7 | -5.4, | 8.4 | -5.6 | -12.2, | 1.1 |
| *Arm 4* | -2.9 | -9.3, | 3.6 | -2.8 | -9.0, | 3.7 |
| **Other foods^b^** |  |  |  |  |  |  |
| *Arm 2* | -1.1 | -2.4, | 0.2 | 0.1 | -0.9, | 1.2 |
| *Arm 3* | -0.5 | -1.6, | 0.6 | -0.0 | -1.0, | 0.9 |
| *Arm 4* | -0.1 | -1.6, | 1.4 | 0.1 | -1.1, | 1.2 |
| **Savoury snacks^b^** |  |  |  |  |  |  |
| *Arm 2* | -0.6 | -1.2, | 0.1 | -0.0 | -0.7, | 0.7 |
| *Arm 3* | -0.3 | -1.0, | 0.5 | 0.1 | -0.5, | 0.8 |
| *Arm 4* | -0.1 | -0.9, | 0.7 | -0.3 | -0.9, | 0.3 |
| **Sweet snacks^b^** |  |  |  |  |  |  |
| *Arm 2* | -0.3 | -1.0, | 0.3 | 0.2 | -0.5, | 0.9 |
| *Arm 3* | -0.1 | -0.9, | 0.8 | 0.1 | -0.6, | 0.8 |
| *Arm 4* | -0.5 | -1.2, | 0.3 | 0.3 | -0.4, | 1.0 |
| **Alcoholic drinks^c^** |  |  |  |  |  |  |
| *Arm 2* | -2.8 | -8.0, | 2.7 | -0.1 | -4.5, | 3.9 |
| *Arm 3* | -2.9 | -7.9, | 2.2 | -0.1 | -4.3, | 3.7 |
| *Arm 4* | -0.5 | -6.1, | 5.2 | -0.5 | -4.4, | 3.4 |

The number of shoppers within each food group is presented in Table 2.

^a^ Based on 10,000 non-parametric bootstrap replicates; Bold numbers represent statistically significant results.

^b^ Percentage was calculated based on the total grams purchased (considering the lack of healthy products in these food groups) excluding the grams purchased from alcohol

^c^ Percentage was calculated based on the total grams purchased

# Supplementary Table 8. Mean differences (98% CI)^a^ in the grams healthy and grams unhealthy purchased within food groups in Arm 2 (information nudge), Arm 3 (position nudge), and Arm 4 (information and position nudges) compared to arm 1, by area-level deprivation

|  | **Grams healthy** | | | | | | **Grams unhealthy** | | | | | |
| --- | --- | --- | --- | --- | --- | --- | --- | --- | --- | --- | --- | --- |
|  | **Deprived areas** | | | **Non-deprived areas** | | | **Deprived areas** | | | **Non-deprived areas** | | |
|  | **Mean diff.** | **98%** | **CI** | **Mean diff.** | **98%** | **CI** | **Mean diff.** | **98%** | **CI** | **Mean diff.** | **98%** | **CI** |
| **Fruits** |  |  |  |  |  |  |  |  |  |  |  |  |
| *Arm 2* | 126 | -222, | 533 | **-366** | **-691,** | **-76** | 59 | -209, | 351 | -129 | -435, | 181 |
| *Arm 3* | -79 | -346, | 146 | **-331** | **-632,** | **-44** | 60 | -203, | 360 | -48 | -371, | 292 |
| *Arm 4* | -14 | -301, | 270 | **-352** | **-653,** | **-82** | -8 | -229, | 218 | 53 | -284, | 382 |
| **Vegetables** |  |  |  |  |  |  |  |  |  |  |  |  |
| *Arm 2* | 271 | -28, | 621 | -263 | -584, | 55 | 20 | -160, | 222 | -37 | -169, | 100 |
| *Arm 3* | 119 | -133, | 371 | -212 | -527, | 86 | -108 | -269, | 47 | 35 | -97, | 167 |
| *Arm 4* | **249** | **2,** | **488** | -122 | -449, | 198 | -13 | -189, | 167 | 85 | -68, | 247 |
| **Breads** |  |  |  |  |  |  |  |  |  |  |  |  |
| *Arm 2* | 40 | -278, | 377 | -224 | -590, | 63 | 38 | -136, | 210 | -148 | -440, | 150 |
| *Arm 3* | -41 | -334, | 231 | 74 | -300, | 393 | 105 | -74, | 292 | -145 | -428, | 176 |
| *Arm 4* | -39 | -299, | 226 | -15 | -362, | 268 | 168 | -71, | 406 | -163 | -445, | 104 |
| **Bread substitutes** | |  |  |  |  |  |  |  |  |  |  |  |
| *Arm 2* | 1 | -58, | 59 | -40 | -130, | 38 | 25 | -70, | 120 | -2 | -112, | 118 |
| *Arm 3* | 7 | -51, | 67 | -55 | -140, | 19 | 38 | -59, | 145 | -25 | -127, | 74 |
| *Arm 4* | 29 | -36, | 95 | -58 | -150, | 18 | -24 | -126, | 87 | 76 | -86, | 287 |
| **Potatoes** |  |  |  |  |  |  |  |  |  |  |  |  |
| *Arm 2* | 219 | -101, | 540 | 144 | -133, | 430 | 63 | -131, | 272 | 49 | -115, | 206 |
| *Arm 3* | 9 | -293, | 291 | 158 | -90, | 416 | -4 | -145, | 130 | 49 | -126, | 218 |
| *Arm 4* | 8 | -302, | 286 | 108 | -139, | 354 | 50 | -120, | 241 | 107 | -113, | 354 |
| **Pasta and rice** |  |  |  |  |  |  |  |  |  |  |  |  |
| *Arm 2* | 11 | -284, | 331 | 121 | -36, | 298 | -68 | -361, | 182 | 2 | -142, | 148 |
| *Arm 3* | 87 | -243, | 380 | -24 | -169, | 124 | **-231** | **-515,** | **-1** | -13 | -173, | 157 |
| *Arm 4* | -1 | -291, | 275 | 63 | -85, | 244 | **-274** | **-553,** | **-42** | -52 | -187, | 78 |
| **Teas and coffees** |  |  |  |  |  |  |  |  |  |  |  |  |
| *Arm 2* | -7 | -177, | 170 | 1 | -85, | 91 | 44 | -62, | 148 | -246 | -605, | 21 |
| *Arm 3* | -15 | -200, | 162 | 6 | -101, | 113 | 54 | -83, | 189 | -206 | -570, | 92 |
| *Arm 4* | -34 | -201, | 108 | 31 | -75, | 146 | 2 | -145, | 151 | -273 | -634, | 16 |
| **Sodas, waters and juices** |  |  |  |  |  |  |  |  |  |  |  |  |
| *Arm 2* | -1209 | -9082, | 7133 | **15051** | **450,** | **43388** | -318 | -1680, | 1022 | 154 | -1242, | 1544 |
| *Arm 3* | -6078 | -12790, | 436 | 4604 | -1200, | 12099 | 786 | -470, | 2165 | -650 | -1847, | 449 |
| *Arm 4* | -1830 | -9403, | 6094 | -243 | -4837, | 4649 | -398 | -1701, | 810 | -664 | -2054, | 717 |
| **Cheeses** |  |  |  |  |  |  |  |  |  |  |  |  |
| *Arm 2* | 35 | -22, | 99 | 6 | -49, | 77 | -23 | -131, | 90 | **-76** | **-151,** | **-3** |
| *Arm 3* | 13 | -47, | 87 | **55** | **6,** | **108** | -14 | -113, | 73 | -5 | -96, | 87 |
| *Arm 4* | 45 | -28, | 134 | **51** | **4,** | **98** | -6 | -116, | 100 | 45 | -47, | 146 |
| **Milk and yogurt products** |  |  |  |  |  |  |  |  |  |  |  |  |
| *Arm 2* | 580 | -628, | 2032 | -100 | -751, | 628 | 322 | -383, | 1295 | -75 | -622, | 459 |
| *Arm 3* | 119 | -816, | 1054 | -138 | -883, | 671 | 138 | -382, | 738 | 35 | -476, | 547 |
| *Arm 4* | 139 | -768, | 1022 | -183 | -943, | 593 | -61 | -505, | 357 | 47 | -451, | 551 |
| **Meats** |  |  |  |  |  |  |  |  |  |  |  |  |
| *Arm 2* | 92 | -33, | 228 | -43 | -161, | 73 | 22 | -133, | 201 | 18 | -98, | 129 |
| *Arm 3* | 74 | -39, | 208 | -21 | -129, | 83 | 9 | -118, | 133 | 54 | -61, | 169 |
| *Arm 4* | 36 | -58, | 131 | -45 | -163, | 65 | 97 | -45, | 248 | **128** | **1,** | **258** |
| **Fish** |  |  |  |  |  |  |  |  |  |  |  |  |
| *Arm 2* | 49 | -80, | 196 | -63 | -148, | 27 | 101 | -66, | 300 | 3 | -198, | 199 |
| *Arm 3* | 8 | -69, | 86 | -24 | -110, | 60 | -29 | -127, | 67 | -71 | -250, | 68 |
| *Arm 4* | 62 | -50, | 186 | 14 | -80, | 117 | -18 | -127, | 86 | 47 | -136, | 261 |
| **Legumes** |  |  |  |  |  |  |  |  |  |  |  |  |
| *Arm 2* | 39 | -221, | 301 | -87 | -305, | 121 | -58 | -313, | 176 | -37 | -225, | 134 |
| *Arm 3* | -22 | -256, | 202 | -96 | -322, | 107 | -152 | -403, | 87 | 42 | -191, | 289 |
| *Arm 4* | 8 | -246, | 244 | 5 | -264, | 279 | -50 | -341, | 250 | -31 | -244, | 181 |
| **Nuts** |  |  |  |  |  |  |  |  |  |  |  |  |
| *Arm 2* | 12 | -92, | 115 | -23 | -88, | 42 | 35 | -40, | 113 | -28 | -149, | 67 |
| *Arm 3* | 16 | -91, | 137 | -7 | -80, | 72 | 79 | -10, | 175 | -8 | -115, | 82 |
| *Arm 4* | -19 | -97, | 55 | 24 | -61, | 127 | 65 | -16, | 154 | -30 | -145, | 68 |
| **Fats** |  |  |  |  |  |  |  |  |  |  |  |  |
| *Arm 2* | 29 | -123, | 173 | -88 | -277, | 77 | 51 | -58, | 165 | 23 | -99, | 145 |
| *Arm 3* | -22 | -160, | 111 | -17 | -223, | 186 | 5 | -93, | 100 | 3 | -101, | 103 |
| *Arm 4* | -18 | -167, | 128 | -26 | -192, | 124 | -61 | -157, | 38 | -2 | -126, | 127 |
| **Other foods** |  |  |  |  |  |  |  |  |  |  |  |  |
| *Arm 2* | NA |  |  | NA |  |  | -51 | -300, | 193 | -160 | -370, | 42 |
| *Arm 3* | NA |  |  | NA |  |  | -73 | -302, | 159 | **-196** | **-376,** | **-27** |
| *Arm 4* | NA |  |  | NA |  |  | -76 | -302, | 140 | -154 | -378, | 89 |
| **Savoury snacks** |  |  |  |  |  |  |  |  |  |  |  |  |
| *Arm 2* | NA |  |  | NA |  |  | -24 | -161, | 111 | 42 | -98, | 186 |
| *Arm 3* | NA |  |  | NA |  |  | -66 | -167, | 37 | -14 | -140, | 107 |
| *Arm 4* | NA |  |  | NA |  |  | -32 | -159, | 94 | 12 | -124, | 147 |
| **Sweet snacks** |  |  |  |  |  |  |  |  |  |  |  |  |
| *Arm 2* | NA |  |  | NA |  |  | -11 | -148, | 135 | -43 | -175, | 87 |
| *Arm 3* | NA |  |  | NA |  |  | 61 | -171, | 439 | -89 | -227, | 41 |
| *Arm 4* | NA |  |  | NA |  |  | -78 | -212, | 61 | 23 | -156, | 206 |
| **Alcoholic drinks** |  |  |  |  |  |  |  |  |  |  |  |  |
| *Arm 2* | NA |  |  | NA |  |  | 71 | -2379, | 2998 | 632 | -1725, | 3050 |
| *Arm 3* | NA |  |  | NA |  |  | -152 | -2103, | 1890 | 566 | -1604, | 2444 |
| *Arm 4* | NA |  |  | NA |  |  | 2457 | -863, | 6446 | 700 | -1472, | 2770 |

The number of shoppers within each food group is presented in Table 2; NA: Not applicable.

^a^ Based on 10,000 non-parametric bootstrap replicates; Bold numbers represent statistically significant results.
